# Acceptability of blinded continuous glucose monitoring for people with serious mental illness and type 2 diabetes: findings from an exploratory mixed-methods study

**DOI:** 10.3389/fcdhc.2026.1923186

**Published:** 2026-10-07

**Authors:** Jennifer V.E. Brown, Ramzi A. Ajjan, Najma Siddiqi, Peter A. Coventry

**Affiliations:** 1 School of Medicine and Population Health, University of Sheffield, Sheffield, United Kingdom; 2 Department of Health Sciences, University of York, York, United Kingdom; 3 Leeds Institute of Cardiovascular and Metabolic Medicine, University of Leeds/Leeds Teaching Hospitals NHS Trust, Leeds, United Kingdom; 4 Hull York Medical School, University of York, York, United Kingdom & Bradford District Care NHS Foundation Trust, Saltaire, United Kingdom; 5 School of Nursing and Public Health, Manchester Metropolitan University, Manchester, United Kingdom

**Keywords:** acceptability, feasibility, mixed-methods, serious mental illness (SMI), type 2 diabetes, continuous glucose monitoring (CGM)

## Abstract

**Introduction:**

People with serious mental illness (SMI) experience a disproportionately high prevalence of type 2 diabetes and a reduced life expectancy of 15 to 20 years. While continuous glucose monitoring (CGM) has revolutionised diabetes self-management in the general population, its feasibility and acceptability among individuals living with co-existing SMI remain largely unexamined.

**Methods:**

The study was embedded within the DIAMONDS feasibility study. Community-dwelling adults with SMI and type 2 diabetes (N = 21 eligible) were invited to wear a blinded CGM sensor (FreeStyle LibrePro) for 14 days. Quantitative metrics evaluated uptake, wear duration, data completeness, and adverse events. Semi-structured qualitative interviews (n = 9) explored user experiences. The qualitative analysis used a hybrid human-AI framework approach to map findings onto the Theoretical Framework of Acceptability (TFA) and the Theoretical Domains Framework (TDF), followed by data integration using a convergence coding matrix.

**Results:**

Sixteen participants agreed to wear the device (76.2% uptake), achieving a mean wear duration of 11 days. Of the returned sensors with data, 92.8% captured ≥5 days of readings, and no adverse events were reported. Qualitative analysis demonstrated highly positive affective attitudes and a significantly lower perceived burden compared with traditional finger-stick testing, despite minor challenges with sensor adhesion. Cross-framework integration revealed that high acceptability was driven by the device’s low-friction physical layout, positive participant beliefs about data consequences, and alignment with a proactive, research-contributing identity.

**Discussion:**

The use of blinded CGM is both feasible and acceptable for people living with SMI and type 2 diabetes. These findings offer a robust foundation for developing structured, low-burden, and theory-informed physical health interventions aimed at closing the mortality gap in this underserved population.

## 1 Introduction

People with serious mental illness (SMI), conditions like schizophrenia, psychosis, or bipolar disorder, are two to three times more likely to have type 2 diabetes compared with the general population (1–3). They also experience worse outcomes and more diabetes complications that tend to be more serious than in the general population without SMI (4). In combination with other physical long-term conditions that are also more common in people with SMI, diabetes contributes to the so-called “mortality gap”, i.e. the reduction in life expectancy by about 15 to 20 years among people with SMI compared with the general population (5). The reasons for the increased risk are complex and varied and include societal factors, medication side effects, and the impact of SMI symptoms (6). Additionally, there is evidence that people with SMI find it challenging to engage with the healthcare system which leads to delayed diagnosis and inconsistent care (7, 8).

For many people with diabetes, blood glucose monitoring is a crucial part of their care and integral to their self-management regimen. Traditionally, this has been done through clinic-based blood tests, supplemented with self-monitoring of blood glucose (SMBG) using fingerstick tests. Continuous glucose monitoring (CGM) has revolutionised glucose monitoring by offering a body-worn, minimally invasive alternative that provides a greater amount of more accurate data. CGM systems involve a sensor that is affixed to the upper arm or abdomen and measures interstitial glucose levels, recording between 198 and 1440 measurements a day, depending on the model (9). These levels can either be accessed in real time by the wearer using their smartphone (unblinded systems) or they are downloaded after the end of the wear period (typically ten to 14 days) using a specifically designed reader (blinded systems).

In the UK, as in other countries around the world, uptake of CGM in diabetes care is increasing all the time. However, access to the technology varies depending on local licensing, insurance, and funding arrangements. The rapid rollout has primarily been driven by the simplification of the care pathway that has been made possible by CGM as well as an increasing body of evidence supporting the effectiveness of CGM in managing blood glucose levels and increasing time-in-range. i.e. time during which blood glucose levels are within a pre-specified target range (10). While most of the evidence is based on individuals with type 1 diabetes, there is a growing evidence base suggesting that people with type 2 can benefit considerably, too, especially when CGM is used as part of a wider package for behaviour modification (11, 12).

Since 2022, people in the UK living with type 1 diabetes have been eligible to receive CGM without cost at the point of care as part of NHS care. As per guidance issued by the National Institute for Health and Care Excellence (NICE), this applies to everyone with this diagnosis, regardless of age or location (NICE guidance 17) (13). In parallel, CGM is being made available to certain groups of people with type 2 diabetes who are receiving insulin treatment, including those with learning disabilities, and impaired glycaemic awareness (NICE guidance 28) (14). Despite their increased risk and worse outcomes, people with SMI are not currently automatically eligible for CGM, unless they meet the other criteria stipulated.

Globally, CGM eligibility criteria and funding arrangements vary widely, with access to the technology frequently subject to health insurance coverage. The American Diabetes Association (ADA) and the European Association for the Study of Diabetes (EASD) highlight the importance of CGM for glucose management, but implementation is variable (15, 16). For example, CGM is available to Medicare recipients who have type 1 diabetes and can evidence at least four fingerstick blood glucose tests per day. Eleven out of the 36 US states where Medicaid coverage includes CGM follow the same approach, with considerable variation in the other states. For people with type 2 diabetes there is even more variability in access (17). Australia’s approach is similar to that of the UK (18) and in India, there is no national approach to eligibility and CGM adoption is limited by cultural, economic, clinical and infrastructure concerns (19). Reliable information regarding national approaches to CGM access and eligibility in African countries is difficult to obtain. None of the available sources internationally give specific consideration to people with SMI.

In the general population with diabetes, modern CGM systems have been found to be highly acceptable, with high treatment satisfaction, improved diabetes management and understanding, and better quality of life. Most of the contemporary evidence is based on the unblinded systems that are widely licensed for clinical use. However, blinded (or professional) CGM, which allow for blinded data to be reviewed by a healthcare professional following a wear period have also been found to be effective (20, 21). Due to restrictive eligibility criteria and/or unclear reporting of (mental) comorbidities, it is unclear whether these findings are applicable to people with SMI and diabetes. Caution is needed here as it is understood that people with SMI interact differently with healthcare (7, 8) and with technology than the general population (22–24). Therefore, acceptability and feasibility among people with SMI is unknown and specific evidence about CGM in people with SMI is needed.

The study presented here forms part of a larger programme of work aiming to develop a structured CGM intervention for people with SMI that is designed to address their needs and circumstances (25). The definitions of the concepts “acceptability” and “feasibility” used in this study were informed by Sekhon’s Theoretical Framework of Acceptability (TFA) (26). In simplified terms, the question about “acceptability” was concerned with user experience and treatment satisfaction (“Do they like it?”), while “feasibility” was about the practicalities of asking people with SMI to use CGM (“Can it be done?”).

### 1.1 Aims and objectives

The aims of this study were

1. To assess if it is feasible to use blinded CGM with people with SMI and type 2 diabetes.
2. To assess the acceptability of blinded CGM to people with SMI and type 2 diabetes.

We addressed these aims through the following objectives:

Objective 1: To collect quantitative data about uptake, wear period, and completeness of data collection in a sample of people with SMI and type 2 diabetes taking part in the DIAMONDS feasibility study.

Objective 2: To undertake qualitative interviews with participants and their informal carers about their experience of using CGM and taking part in the study to identify potential barriers and enablers of CGM use in people with SMI.

## 2 Materials and methods

The study presented here was embedded in the context of the feasibility study for the new DIAMONDS intervention (27). Wearing a sensor was not part of the intervention and participants were invited to try CGM after completion of the DIAMONDS intervention phase.

### 2.1 Participants and recruitment

Between March and June 2022, we worked with individuals taking part in the DIAMONDS feasibility study (ISRCTN: 15328700) which is reported in detail elsewhere (27, 28). Briefly, this single-group study recruited community-dwelling adults aged 18 or over with confirmed diagnoses of a serious mental illness (defined as schizophrenia, schizoaffective disorder, psychosis, or bipolar disorder) and type 2 diabetes from six NHS mental health trusts in the North of England. Individuals were eligible to take part regardless of their diabetes treatment or glucose monitoring regimen, i.e., insulin-treated and non-insulin-treated type 2 diabetes were eligible. Individuals with type 1 diabetes or diabetes due to an underlying condition, inpatients, and those lacking capacity to consent (under the Mental Health Act) were not eligible to take part. After baseline data collection was completed, participants were paired with a trained intervention facilitator, called a DIAMONDS Coach, to receive 16-weeks of self-management support for their physical and mental health. The development and components of the DIAMONDS intervention are described elsewhere (29). Participants continued received all ongoing mental health and diabetes care and continued any established diabetes management approaches throughout the study.

We also intended to recruit informal/family carers to take part in qualitative interviews about their experience of the CGM wear period. Participants were asked to identify any informal/family carers who support them to manage their health.

### 2.2 Data download and analysis

Data were downloaded using LibreView software (web based). To assess feasibility, the following descriptive information was recorded:

- Uptake (number of participants agreeing to wear a sensor; n/N, %)
- Sensor loss (number of sensors not returned; n/N, %)
- Duration of data collection (wear period in days; mean, SD, median, range)
- CGM data availability (number of participants with at least five or ten days of data collection; n/N, %)
- Premature sensor loss (number of participants with sensor detachment before 14 days; n/N, %)
- Adverse events

O Total number of adverse events reported.

O Number of participants with any adverse event (n/N, %).

In addition, details about instances of sensor and data loss, and about adverse events were recorded narratively. Adverse events were defined as any untoward or negative response or symptoms during the sensor wear period, including (but not limited to), skin problems at the sensor site (e.g., redness, swelling, allergic reaction to the sensor adhesive), and mental health problems (e.g., anxiety related to the sensor). Participants were asked to contact the local study team to report adverse events and they had the opportunity to discuss any issues during the sensor removal appointment. The qualitative interviews offered a chance to discuss adverse events in more depth.

In line with international recommendations (30), interstitial glucose readings were summarised descriptively using the following measures:

- Percentage time in, below, and above range 3.9 to 10.0 mmol/L (mean, SD)
- Glycaemic variability (% coefficient of variation; mean, SD), target ≤36%
- Glucose management indicator (mmol/L as generated by the LibreView software based on the data recorded by the sensor)

In addition, the following characteristics were recorded:

- Gender
- Ethnicity
- Age (years; mean, SD)
- BMI (kg/m^2^; mean, SD)
- Baseline HbA1c (mmol/L; mean, SD)
- Diabetes treatment

### 2.3 Qualitative interviews

Information relating to the qualitative component of this study is reported in line with COREQ (consolidated criteria for reporting qualitative research). A completed COREQ checklist is included in appendix 1.

#### 2.3.1 Participant recruitment

To inform our evaluation of the acceptability of CGM to individuals with SMI and type 2 diabetes and their carers, we conducted semi-structured qualitative interviews. Towards the end of the 14-day wear-period, the lead investigator (JB) contacted participants and invited them to give feedback about their experience of using the sensor as well as their use of technology more generally to support their health and wellbeing. All participants who were offered a CGM sensor were eligible to be invited for an interview (regardless of whether they agreed to wear the sensor or not) and were initially sent an interview information pack, including an invitation letter, participant information sheet, and consent form (see appendix 2). Participants were then contacted via telephone, text message, or email (depending on previously expressed contact preferences). At this point, participants had the opportunity to ask questions about the interviews. They were also asked if they had a family carer who may be interested in taking part in an interview. For those participants who agreed to take part, a mutually suitable time was agreed for the interview. Interviews could not be conducted in-person because of social distancing restrictions in force during the Covid-19 pandemic. Participants were given a choice to be interviewed by telephone or video call. Before the start of the interview, participants gave verbal informed consent. With their permission, interviews were recorded. Where participants did not agree to a recording, JB took notes during the interview. Notes were anonymised and did not contain any identifiable information. Participants retained their six-digit identification number from the DIAMONDS feasibility study, where the first three digits were the site identification, with the final three digits allocated to consecutively enrolled participants.

#### 2.3.2 Positionality statement

All interviews were carried out by the lead author, JB. JB is a cisgender woman. At the time of the study, JB held an MSc in Health Psychology and was employed as a Research Fellow while also undertaking work towards a PhD in Health Sciences. JB has received training in qualitative research methods and analysis as well as ongoing supervision during her PhD from the co-authors. In addition, JB has experience with the responsible use of generative AI in research. JB is an experienced researcher and has experience in working with people with SMI, including undertaking and analysing qualitative interviews in this population. JB did not have a direct relationship with study participants prior to the interviews. Before the start of the interviews, JB explained the reasons for undertaking the qualitative component of the study and how the interviews will form part of her PhD. While unconscious biases cannot be ruled out, JB approached the interviews without any preconceptions or assumptions about the participants or any expectations about their experience using CGM.

#### 2.3.3 Interview structure

Interviews were semi-structured based on approved topic guides (see appendix 3) with questions designed to explore individual circumstances, experiences, and preferences that may affect acceptability:

1. Self-management routines, including medications, diet, appointments, routines, exercise/activity, social events/contacts
2. Experience of using technology (apps or devices) to support self-management
3. Use of technology to support diabetes self-management
4. Reasons for agreeing (or declining) to wear the CGM
5. Feelings (positive, curious, anxious, nervous etc.) prior to having the CGM fitted compared with feelings now
6. Experience (likes, dislikes, problems) of wearing the CGM (incl. fitting and removal)
7. Thoughts about wearing a CGM again in the future and/or wearing a different (unblinded) model

Interview recordings were transferred securely to a third-party transcription company where they were anonymised (all person and place names removed) and transcribed verbatim. Transcripts and notes from one interview which was not recorded were saved as MS Word documents.

#### 2.3.4 Analysis

Analysis involved two stages. First, we undertook an inductive data-driven coding process that drew on the principles of reflexive thematic analysis (31) and constant comparison (32) to develop themes grounded in participants’ accounts. Second, these themes were examined in relation to existing theoretical frameworks (Theoretical Framework of Acceptability [TFA] (26) and the Theoretical Domains Framework [TDF] (33, 34)), allowing us to interpret the findings through established constructs (35) and to later triangulate qualitative findings with the quantitative data. This sequential and hybrid approach enabled us to retain sensitivity to context while situating the analysis within a broader theoretical and applied evidence base. Our combined approach offered a way to balance theoretical sensitivity with openness to participants’ lived experiences, thereby making sure that the analysis remained both conceptually grounded and responsive to context (36).

The analysis was led by JB and involved the following steps:

1. Familiarisation – Reading and re-reading of transcripts without any intention of coding. Free text notes were made during this stage to capture salient points from each interview.
2. Inductive coding and organisation into descriptive themes – Initial labels were assigned to interview sections to summarise and capture participants’ accounts and experiences. This was done in MS Word for each transcript individually which resulted in a multitude of different labels being used to capture similar content across transcripts. The initial labels were reviewed and refined to produce a final unified list of codes. This was used as the “codebook” during the next phase which involved rereading the transcripts and assigning codes consistently across all transcripts. Working offline to allow immersion in the data, coded transcripts were printed and cut into sections. JB physically sorted quotes from all participants according to their assigned codes. Participant identification numbers were used to organise quotes without revealing participants’ identity. During this process, four descriptive themes were identified.
3. Revision – The final list of codes and the descriptive themes were reviewed and sense checked by PC, confirming the logical structure of the coding framework as analysis progressed. When coding was complete for all transcripts, participant quotes were organised into a coding matrix, where columns represented codes and rows represented participant IDs. During this stage, final refinements were made to the codes and thematic structure, for example by collapsing multiple similar codes into one and/or reassigning codes to a different theme.
4. Deductive framework mapping – To support JB, we developed a custom Generative Pre-trained Transformer (GPT; Gemini Gem; using a paid Gemini Pro subscription with unlimited use of “Thinking” mode).Codes were mapped to TFA and TDF domains in January 2026. The Gem generated initial domain mappings based on codes and illustrative quotes. JB reviewed all mappings, interrogated model outputs where needed, and made final decisions. Gem instructions were iteratively refined to improve consistency, enhance transparency and efficiency, while retaining full researcher oversight of analytic decisions. The GPT mapping was not independently validated by a second human coder. Full details and the final version of the GPT’s instructions are included in appendix 4.
5. Understanding acceptability – quotes and codes mapped against the TFA were analysed separately to allow a detailed understanding of CGM acceptability among people with SMI and type 2 diabetes.
6. Cross-framework mapping – To bridge the conceptual gap between the TFA and the TDF, areas of cross-over between the two frameworks were interrogated and interpreted with the aim of understanding how acceptability maps to psychological determinants of behaviour. We based this approach on previous work undertaken by Rushton et al. (2020) (37). To provide structure to the cross-over of two complex multi-domain frameworks, findings from this step are visualised using a heatmap. By tabulating the TDF domains (columns) against the domains of the TFA (rows), the heatmap shows the number of codes for each intersection between the two frameworks, for example the number of codes mapped against *Beliefs about consequences* (TDF) and *Affective attitude* (TFA).

### 2.4 Integration of qualitative and quantitative findings

To integrate the quantitative findings about sensor uptake and data completeness with the qualitative data from the interviews, we followed a triangulation protocol (38). The aim was to synthesise the findings from the two phases of the study under the domain headings of the TFA, summarising what both the qualitative and the quantitative dataset showed about the acceptability and feasibility of CGM in this context. To illustrate the triangulated findings, we used a convergence coding matrix (39), i.e. a table that shows areas of congruence (agreement), dissonance (disagreement), or silence between the qualitative and quantitative findings for each of the seven domains of the TFA. Where applicable, we indicated partial congruence or dissonance. “Silence” was used to show where only one type of data provided information relating to a TFA domain.

The convergence coding matrix was developed by JB after the completion of the descriptive quantitative analyses and the qualitative framework analysis based on the TFA. PC reviewed the initial matrix; refinements to the matrix and the integrated interpretation of the findings were made in collaboration between PC and JB.

### 2.5 Service-user and carer involvement

Consultation and discussion with the service user and carer group DIAMONDS Voice contributed to the decision to include the offer to wear a CGM in the DIAMONDS feasibility study. We discussed with the group that this had not previously been done, that the devices would be blinded, and that there would be no guarantee that participants would be able to access comparable technology as part of their usual diabetes care upon completion of the study. DIAMONDS Voice members agreed that it was important to take this opportunity to offer innovative technology to a population that often misses out in the interest of informing future research and, ultimately, healthcare. There was consensus between the study team and DIAMONDS Voice that clear communications about the functionalities of the sensor would be key, with a focus on specific concerns people with SMI might have. For example, participants experiencing delusions or hallucinations might have required reassurance that the sensor would not track their location or activity or transmit any personal data.

Consequently, DIAMONDS Voice co-designed the participant-facing documentation (information materials and consent forms) for this study, as well as the wording of the information that was provided to participants during their CGM fitting appointment. DIAMONDS Voice reviewed draft topic guides for the interviews and commented on the content and style of the questions to ensure that they were worded clearly and in such a way that would allow the participant the opportunity to elaborate freely on their answer. Questions that were deemed to be ambiguous were simplified and prompts were included in the topic guide where needed.

## 3 Results

### 3.1 Quantitative findings

#### 3.1.1 Description of the sample

**Table 1** below summarises demographic information about the sample as well as key CGM measures.

**Table 1:** Demographic participant characteristics and key CGM measures.

Participant characteristics	Total sample	Accepted CGM	Declined CGM
N	21	16	5
Gender, n/N, (%) female	9/21 (43)	6/16 (37)	3/5 (60)
Ethnicity, n/N (%)			
Asian Indian/Asian Pakistani	3/21 (14)	1/16 (6)	2/5 (40)
Black/Other	3/21 (14)	2/16 (12)	1/5 (20)
White	15/21 (72)	13/16 (82)	2/5 (40)
Age, years (Mean +/- SD)	50.7 +/- 8.1	51.0 +/- 6.9	50.0 +/- 10.8
Body mass index, kg/m	36.9 +/- 8.7	37.5 +/- 9.3	35.3 +/- 6.1
Baseline HbA1c, mmol/mol (Mean +/- SD)	66.0 +/- 14.0	68.0 +/- 14.1	61.0 +/- 11.2
Diabetes treatment [n/N (%) receiving insulin]	5/21 (24)	4/16 (25)	1/5 (20)
CGM measures			
Glucose management indicator, mmol/mol (mean +/- SD)	-	61.9 +/- 14.6	-
Percent time in range (Mean +/- SD)	-	56.1 +/- 28.1	-
Percent time below range (Mean +/- SD)	–	4.8 +/- 8.0	–
Percent time above range (Mean +/- SD)	–	39.2 +/- 32.7	–
Glucose variability (%, mean +/- SD)	-	26.7 +/- 7.5	-

#### 3.1.2 Uptake

Between July and December 2021, 30 participants were recruited to take part in the DIAMONDS feasibility study (27). Nine withdrew before the end of the intervention delivery period (March to June 2022); 21 individuals were eligible to be offered the FreeStyle Libre Pro CGM. Sixteen (76.2%) accepted and five declined.

#### 3.1.3 CGM device and procedures

At the end of the 16-week intervention period, participants were invited to wear a blinded CGM sensor, a FreeStyle LibrePro (manufactured by Abbot), for 14 days. Participants had provided consent to being offered a CGM when they joined the study, however, participants were free to decline at the point of invitation without having to provide a reason. Participants who accepted the invitation to wear the LibrePro met with a trained healthcare professional (HCP) to have the sensor fitted on the back of their upper arm and then activated to initiate data collection. Information about the sensor was provided to the participant at this stage.

Participants were instructed to wear the device for 14 days (two weeks) and not to attempt to remove it themselves. Instead, an appointment was arranged with a trained HCP to remove the device. After the completed wear period, HCPs were asked to return the sensors to the study team at the University of York.

##### 3.1.3.1 Sensor loss

Twenty-one sensors were used with the 16 participants who agreed to take part in this phase of the study;14 were returned with usable data. The other seven sensors were lost/misplaced by participants (n=2), lost by staff (n=3), or discarded without any data being recorded due to problems during fitting (n=2).

Four participants received more than one sensor: One participant received a second sensor after the first one was lost by staff after removal at the end of the wear period. Similarly, one participant agreed to wear a second sensor after the first sensor was lost in the post following the initial wear period. For one participant, the sensor fell off immediately after fitting and a second one could be fitted during the same appointment. With one participant, a total of three sensors were used: the first sensor fell off after four days, a second sensor had to be discarded during fitting as it could not be activated, and a third sensor also fell off within four days.

In the other instances of sensor loss, the participant was not fitted with another sensor.

##### 3.1.3.2 Duration of data collection

All participants were instructed to wear the sensor for 14 days, after which a removal appointment was arranged. The mean sensor wear period was 11 days (SD 4.57 days; median 13 days; range 1 to 15 days).

##### 3.1.3.3 Data completeness

Of the 14 sensors from 16 participants that were returned with data, 13 were worn for five days or more (92.8%). Nine of the 14 (64.3%) were worn for ten days or more.

##### 3.1.3.4 Premature sensor detachment

Of the 16 participants who wore a sensor, two experienced premature sensor loss, i.e. the sensor detached before the end of the 14-day wear-period. In both instances, the participant had more than one sensor fitted but none stayed in place for the full 14-day period.

##### 3.1.3.5 Adverse events

No adverse events (diabetes or mental health related) were reported. Some participants described pre-existing issues like dry skin as interfering with the sensor adhesive, but these were not considered adverse events or problems attributed to the sensor.

### 3.2 Qualitative findings

Ten out of 16 participants took part in an interview, including participants who ended up using more than one sensor due to issues with the adhesive. One participant did not agree to their interview being recorded. Notes were taken during this interview, but these were subsequently removed from the analyses as the notes were not detailed enough to allow inductive coding. The following results are based on transcripts from nine interviews. Interviews were between 5 and 45 minutes in duration, with the majority taking between 20 and 40 minutes. Five participants were women. We did not undertake any interviews with carers because none of the participants identified an informal carer.

#### 3.2.1 Inductive coding and organisation into descriptive themes

**Table 2** below summarises the four descriptive themes and the codes they contain. The coding matrix showing the participant quotes assigned each code is available in appendix 5.

**Table 2:** Descriptive themes and the codes they contain.

Theme	Codes
1 – The context of living with SMI and physical health problems	12: Medication adherence 13: Diabetes is an invisible illness 17: Managing appointments 19: Relationship strain due to SMI 26: Impact of overweight/obesity 46: SMI dominates 48: Experience of healthcare 50: Impact of SMI symptoms/medication
2 – Existing self-management strategies	*Subtheme: Lifestyle* 16: Motivation to make positive lifestyle changes 28: Successful lifestyle changes 31: Use of health tech 32: Aware of the importance of diet 37: Aware of the importance of physical activity *Subtheme: Support networks* 18: Support from family 20: Support from friends 29: Support from professional carers/HCPs 30: Social contacts *Subtheme: Diabetes management* 21: Experience using SMBG 38: Current glucose monitoring *Subtheme: Individual approaches to self-management* 15: Use of available financial resources 53: Faith/religion as a coping strategy
3 – The CGM experience	1: Issues with sensor adhesive 2: Sensor came off prematurely 8: DIY approaches to trying to get the sensor to stick 33: Experience of sensor wear 35: Experience of sensor removal 44: Experience of sensor fitting
4 – Engagement with CGM as a strategy for diabetes management	3: Interested in seeing CGM data 4: Sense of responsibility for the sensor 22: Motivation for trying CGM 23: Feelings about CGM prior to fitting 24: Suggestions for sensor/CGM improvement 25: Interest in unblinded sensor and app 34: Feelings about wearing the sensor in retrospect 36: Potential of unblinded CGM for diabetes management 40: Prior knowledge of CGM 41: Benefits of CGM over SMBG 42: Perception of CGM as a medical device 45: Effect of sensor on sense of self/own identity 47: Understanding of glucose management 49: Reaction from others to CGM

Overall, this initial inductive phase of the qualitative analysis clearly showed that acceptability of CGM needs to be understood within the context of co-existing SMI and type 2 diabetes. The impact of living with SMI was evident across all areas of life with clear knock-on effects for diabetes management. The introduction of CGM did not happen in a vacuum but within the complex interplay of factors that shape the lives of people with SMI.

#### 3.2.2 Deductive framework mapping

Using codes as the unit of analysis, a custom GPT was trained to map codes to the domains of the TFA and the TDF. Participant quotes were used to illustrate each code. The GPT presented all participant quotes for each code drawing from the coding matrix; JB then selected illustrative quotes for each code that captured the depth and breadth of experiences shared by participants. Codes were mapped against framework domains by the custom GPT based on these illustrative quotes. This was done on a code-by-code basis with JB sense checking the GPT’s mapping and interpretation for each code. JB made corrections where needed and iteratively refined the instructions and prompt structure used. Appendix 5 shows the full list of codes mapped against the TFA and TDF domains, along with interpretations for how domains are linked within and between the two frameworks.

##### 3.2.2.1 Framework “gaps”

Four codes were not mapped against any domains in either framework, namely *50 Impact of SMI symptoms/medication; 26 Impact of overweight/obesity; 19 Relationship strain due to SMI;* and*12 Medication adherence*. These codes describe wider contextual and clinical circumstances associated with living with SMI. Although such factors may shape the environment in which CGM is used, they do not represent specific proximal behavioural determinants of CGM use (TDF) or components of its acceptability (TFA) and were therefore not mapped.

Additionally, themes 1, 2 and 4 also included codes not directly related to CGM use. These themes were not mapped to the TFA, as the analysis focused specifically on the acceptability of CGM. However, they were mapped to the TDF where they provided insight into broader, and in some cases less immediate, determinants of behaviour. All remaining codes were mapped to both frameworks to examine how acceptability is associated with behavioural determinants, which forms the primary focus of the analysis presented below.

Seventeen codes from themes 3 and 4 were mapped against the TFA and TDF, covering six of the seven domains of the TFA (all except *Opportunity costs*) and ten of the 14 TDF domains (all except *Optimism; Reinforcement; Goals;* and *Behavioural regulation*). Most codes were mapped against two or more domains from each framework.

#### 3.2.3 TFA framework analysis – understanding CGM acceptability

Given this study’s focus on acceptability of CGM, we undertook a framework analysis of the quotes contained within the codes that were mapped against the TFA. Overall, participants’ experience of using CGM was positive. This is reflected in the mapping of participant quotes against the domains of the TFA, in particular *Affective attitude* and *Burden*, where most of the quotes clustered.

##### 3.2.3.1 Affective attitude

Participants described that they “forgot it [the sensor] was even there” (400103), and that the sensor caused “no side effects, no pain, it’s easy to wear you can take a shower or bath, whatever you want” (400102), indicating high levels of concurrent acceptability. Prospectively, some participants reported feeling “apprehensive at first. I was really thinking it’s going to hurt” (400103), while others felt “curious” (100102) and used their experience of other medical procedures as a benchmark: “No. I’ve got used to needles now, I have a lot of needles.” (200105). One participant in particular shared their excitement about wearing the sensor with people in their social circles: “I said to people I’m going to be wearing this fancy-pants glucose monitor. [ … ] People I told just wanted to see it.” (300100) In retrospect, participants reported feeling “glad I had it done” (400103) and that it was “great to have it [CGM sensor] on” (300103). One participant described how they “miss it already. I keep checking for it.” (100102).

##### 3.2.3.2 Burden

The positive attitudes towards the sensor were somewhat balanced out by reports of sensors falling off due to issues with the adhesive, and the perception that the sensor was not securely attached, leading some participants to worry about losing it, as highlighted in the quotes below:

“No, before it fell off I could tell that it wasn’t sticky.” (200105)

“I slightly knocked the doorframe … knocked my arm on the doorframe and it just fell off. [ … ] It wasn’t even a hard knock, to be honest.” (600100)

Those participants who had experience of self-monitoring of blood glucose (SMBG) clearly described CGM as less burdensome and more convenient than finger prick testing. This account from a participants illustrates this point and highlights their interest in and appreciation of the potential benefits of using unblinded CGM in the future: “Instead of having to do the intrusive jab yourself with a needle several times a day and then take the reading, you could do away with that because you’d just have that little discreet patch which is - it’s not more obtrusive or indeed intrusive than wearing a patch to try and stop you smoking. So it’s no more than like a nicotine patch or something like that.” (200102)

##### 3.2.3.3 Perceived effectiveness

In the context of this study which was not assessing effectiveness and instead was interested exclusively in acceptability and feasibility of blinded sensors, we did not expect participants to talk in detail about the effectiveness of the sensors, and the topic guide did not include questions to this end. Participants demonstrated some understanding of how the sensor worked and how it might support diabetes management. However, there were clear knowledge gaps about the links between blood sugar levels and lifestyle factors like diet or exercise which are not directly attributable to CGM, but which provide important context. For example, one participant stated that they “wish I had known before that the exercise was that was making a bigger difference” (500104).There seemed to be a general understanding that CGM could be “good for me, do you know what I mean, really good” (300103) and that it could help manage their diabetes without any concept of “how”.

##### 3.2.3.4 Ethicality

The ethical alignment of CGM with the participants’ personal value systems was not discussed in detail in the interview but many participants described their motivation to try CGM as wanting to support research and support the study team. For example, one participant noted that the best thing about the CGM was “that I was contributing to research that could come in useful for others” (200102) which was echoed by another participant who said: “I wanted to help with research, but also, I’ve got a problem with blood sugars, so I wanted to help myself, but I also wanted to help them research that as well” (400103).

##### 3.2.3.5 Intervention coherence

Participants had limited knowledge of CGM before taking part in this study and had no in-depth understanding of CGM as an intervention. One participant said “No, never. [ … ] I had never heard of them.” (500104) whereas another had come across them in popular culture: “Yeah, I’ve seen a few people with them on. I wondered what they were. And I never know until [name of a professional dancer] was dancing on [name of televised dance show]; he took his shirt off and he had one, and then it was in social media about it. I thought ‘oh, that’s what that is then’ “(300100).

##### 3.2.3.6 Self-efficacy

In the specific context of this study, the need for participant self-efficacy was limited as there was no interaction with the sensor and fitting and removal were undertaken by HCPs. As such, the interviews did not really touch on self-efficacy. Those participants who experienced issues with the sensor adhesive and/or sensor loss nonetheless continued with repeated sensor wear and experimented with do-it-yourself approaches to improve adhesion One participant shared their approach: “We got the monitor fitted, bandaged it round and I said, that’s going nowhere.” (200105)

#### 3.2.4 Cross-framework mapping

While the TFA analysis reported above provided a theory informed account of the acceptability of CGM, it does not by itself explain how these perceptions relate to the behavioural determinants that help to understand engagement with the sensors. To address this, a cross-framework mapping of TFA and TDF domains was undertaken to examine how identified dimensions of acceptability intersect with determinants of behaviour. The results of this analysis are presented below (**Table 3**).

**Table 3:** Heatmap of the overlap between TFA and TDF domains, indicating the number of codes mapped to each intersection.

TFA domains	Kn	Sk	SPRI	BaCa	Op	BaCo	Re	In	Go	MADP	ECR	SI	Em	BR
Affective attitude	1		5	1		6		1		2	3	1	4	
Burden		2	2	1		3		1		2	7		4	
Perceived effectiveness			1			2					2		1	
Ethicality			2			2							1	
Intervention coherence	1											1		
Opportunity costs														
Self-efficacy		1									1			

Kn, Knowledge; Sk, Skills; SPRI, Social/professional role and identity; BaCa, Beliefs about capabilities; Op, Optimism; BaCo, Beliefs about consequences; Re, Reinforcement; In, Intentions; Go, Goals; MADP, Memory, attention, and decision processes; ECR , Environmental context and resources; SI, Social influences; Em, Emotions; BR, Behavioural regulation. Darker shading indicates stronger overlap, lighter shading indicates weaker overlap between TFA and TDF domains.

Codes cluster around two TFA domains (Affective attitude, Burden) and three TDF domains (Social/professional role and identity, Beliefs about Consequences, Environmental context and resources). In particular, there are three core areas of conceptual overlap that directly address engagement with CGM which are explored below:

##### 3.2.4.1 Exploring links between the burden of diabetes management and contextual factors

The synergy between TDF *Environmental context and resources* and TFA *Burden* illustrates the direct relationship between the material reality of diabetes management and the participant’s perceived effort. In this study, the physical resources – ranging from finger-prick equipment to the CGM sensor and its adhesive – dictated the “work” required by the individual. Participants characterised the material resources of standard monitoring as a significant burden, or “faff,” which the CGM seemed to eliminate. By changing the resource to a discreet, continuous sensor (TDF: *Environmental context and resources*), CGM reduced the perceived effort (TFA: *Burden*), making glucose monitoring “much easier.” Conversely, when the CGM’s own physical resources failed (e.g., poor adhesive or intrusive placement), it created a new burden, requiring compensatory physical labour and psychological effort from the participant to ensure the intervention remained viable.

##### 3.2.4.2 Beliefs about consequences shape and are shaped by affective responses

The relationship between TDF *Beliefs about consequences* and TFA *Affective attitude* describes the transition from cognitive expectation to emotional evaluation. Participants’ initial feelings were shaped by beliefs about physical outcomes; for example, “apprehension” or “nervousness” (TFA) were rooted in the belief that the fitting will hurt (TDF). As the intervention progressed, this shifted toward a positive attitude driven by the belief that the device provides personal and altruistic benefits. Participants expressed “curiosity” and “interest” (TFA) because they believed the data would provide insights into their own blood sugars or contribute to research for the “next generation” (TDF). Ultimately, the retrospective acceptability was solidified when the realised consequences, such as the device being “great” to wear and easy to remove, superseded initial fears, resulting in an emotional state of being “pleased” or “glad” the intervention was undertaken.

##### 3.2.4.3 Sense of identity affects perceptions about CGM

The synergy between TDF *Social/professional role and identity* and TFA *Affective attitude* demonstrates how CGM acceptability was rooted in its alignment with the participant’s sense of self and their perceived role as a contributor to research. CGM was embraced as a means to “help the next generation” or “contribute information” to science. This adoption of a proactive, altruistic identity (TDF) generated a positive attitude characterized by feeling “glad,” “pleased,” or “curious” (TFA). Furthermore, because the device was considered “discreet” and described to not “really affect you in any way,” it seemed to avoid imposing a stigmatised “medical” identity or altering the participant’s core sense of self (TDF), resulting in a retrospective emotional state of being “fine” and comfortable. CGM was acceptable because it facilitated a sense of “freedom” and social utility while integrating into the participant’s life without conflicting with their internal representation of who they are.

Taken together, these areas of overlap highlight how different dimensions of acceptability are shaped by interconnected behavioural determinants. Acceptability of CGM for people living with SMI and type 2 diabetes can be better understood as a multi-dimensional construct shaped by the interaction between the material, cognitive, and social determinants of behaviour. At its core, acceptability seemed to function through a low friction pathway where the physical reliability of the device (TDF: Environmental context and resources) directly dictated the perceived effort (TFA: Burden) required to maintain the intervention. This material experience was then processed through a cognitive-emotional lens, where beliefs about the data’s ability to be beneficial or to provide insights (TDF: Beliefs about consequences) transformed initial apprehension into a positive emotional response (TFA: Affective attitude). Finally, the intervention’s high degree of acceptability was solidified by its alignment with the participant’s identity; by being discreet and facilitating a role as a contributor to research (TDF: Social/professional role and identity), CGM avoided the stigma of further medicalisation and reinforced a sense of self-worth and satisfaction (TFA: Affective attitude). Synthesis of quantitative and qualitative evidence.

### 3.3 Integration of qualitative and quantitative evidence

Using a convergence coding matrix structured around the domains of the TFA we integrated quantitative and qualitative findings. **Table 4** shows a consistent pattern across key domains of acceptability, alongside notable gaps where quantitative data were not able to capture participants’ experiences.

**Table 4:** Convergence coding matrix.

TFA domains	Congruence dissonance silence	Comments
Affective attitude	Congruence	Participants reported positive attitudes about CGM which is reflected in the high rate of uptake and high levels of data completeness.
Burden	Congruence	The burden of issues with the adhesive was described in interviews and evident in the need for several participants to try multiple sensors.
Intervention coherence	Silence	Described in interviews but not supported in quantitative data.
Self-efficacy	Congruence	Participants’ self-efficacy regarding CGM was described in interviews and demonstrated in their willingness to try repeated wear periods.
Ethicality	Silence	Described in interviews but not supported in quantitative data.
Perceived effectiveness	Silence	Described in interviews but not supported in quantitative data.

There was clear convergence between qualitative and quantitative findings for *Affective attitude, Burden*, and *Self-efficacy*. This suggests that participants’ positive accounts of CGM were reflected in observable engagement behaviours, including high uptake, sustained wear, and self-directed willingness to persist using the devices despite challenges. By contrast, domains related to *Ethicality, Intervention coherence*, and *Perceived effectiveness* were supported by qualitative accounts, but quantitative data was relatively silent. This might be reflective of the limited sample size and possibly the limits of capturing nuanced aspects of acceptability using quantitative feasibility metrics.

By combining qualitative and quantitative data, we have gained a more in-depth and robust understanding of the acceptability and feasibility of CGM in this population. The quantitative data alone might have suggested good uptake, but we would not have understood the reasons behind this. Similarly, the quantitative data about at times repeated engagement with the sensor gives on objective confirmation of the participants’ accounts about how much they enjoyed wearing the CGM sensor. In combination, and despite the small sample size, there is a clear signal here that CGM is acceptable and feasible for people with SMI.

## 4 Discussion

### 4.1 Summary of findings

Our study showed that it is feasible to use CGM with people who have SMI and type 2 diabetes. Additionally, although some participants experienced minor complications, CGM also appears to be acceptable to this population. By integrating quantitative and qualitative findings, using recognisable and widely used theoretical frameworks, our study makes an important and novel contribution to the scientific understanding of physical health management in people with SMI. Importantly, our qualitative findings highlighted the importance of considering diabetes care within the context of living with SMI, and the complex challenges this entails. To the best of our knowledge, at the time of writing this was among the first studies to specifically work with this population, giving access to innovative technology to a small sample of people with a particularly high illness burden who are traditionally excluded from research opportunities (40–43). A mixed-methods study undertaken by Hansen et al. in 2025 reported similar findings to ours, working within the setting of a specialist diabetes service in Denmark (44).

### 4.2 Implications for intervention design and implementation

Hansen et al. (2025) recruited participants from the Fusion Clinic in Denmark, a highly specialised clinic that offers diabetes care to people with SMI (45). Participants were patients at the clinic with regular attendance over the previous six months. CGM was offered to them as part of their ongoing diabetes care and they were then interviewed about their experience (44). Our findings presented here offer complementary evidence that people who are not already enrolled in specialist diabetes care, and indeed receive very little diabetes support beyond annual physical health checks in primary care, are also willing to engage with CGM technology. Participants in our study were recruited from mental health settings, and our previous work has shown that this group generally does not receive appropriate diabetes care or engage with self-management (46). With a view to future implementation in a UK context, where specialist services like the Fusion Clinic do not exist, our study offers important findings about a different population in addition to the evidence reported by Hansen and colleagues.

By mapping qualitative data not only against the TFA but also the TDF, our study has laid the groundwork for future intervention design. The TDF domains form part of the Mechanism of Action (MoA) framework and (47), using the Theory and Techniques Tool (TTT) (48), the domains can be mapped directly against Behaviour Change Techniques (BCTs) (49). The TTT summarises the evidence base for all BCTs, thereby streamlining the development of theory- and evidence-based interventions. In our study, acceptability of the intervention (driven by the TFA domains *Affective attitude* and *Burden*) intersected with determinants of behaviour captured by the TDF domains *Social/professional role & identity*, *Beliefs about consequences*, and *Environmental Context and Resources* which correspond directly to the MoAs of the same names. *Beliefs about consequences* and *Environmental context and resources* are highly modifiable behavioural determinants; the TTT suggests links to several BCTs that have been shown to affect the corresponding MoAs. Future intervention development should consider these BCTs and develop intervention components accordingly, ideally in close collaboration with service users, carers, clinicians, and other stakeholders. Ultimately, when the device is low-burden, high-insight, and identity-preserving, it has the potential to move beyond being a mere clinical tool to become a highly acceptable component of the participant’s life context. Acceptability and feasibility are recognised as key factors to consider in early intervention development, and as key drivers of the implementability and scalability of new interventions (50). Our study has also highlighted the need for appropriate training of healthcare professionals supporting people with SMI to use CGM, and for careful integration of implementation challenges as part of future intervention development. In this study, we asked Research and Development (R&D) staff in NHS mental health trusts to fit and remove the CGM sensors. R&D teams were involved in the recruitment of participants for the DIAMONDS feasibility study but did not have specialist diabetes or CGM training. We provided training as part of our study procedures but issues during fitting and the disposal of several sensors before data could be downloaded for analysis indicate that careful consideration will need to be given to where CGM might best fit into the care pathway in primary care, secondary diabetes care, or secondary mental health services. While staff perspectives were not captured qualitatively in this study, mapping the rich interview data from the participants to the TDF allows initial insights into implementation challenges which future studies should explore in more depth, for example through mapping relevant TDF domains to the Consolidated Framework for Implementation Research (CFIR) (51).

### 4.3 Methodological innovation

Generative AI, predominantly GPTs, are increasingly used to support research. Particularly for qualitative analysis, there has been some controversy about the appropriateness of using such tools (52, 53). In this study, we developed and adopted a hybrid approach that retained a strong “human in the loop” element throughout. By undertaking the initial inductive coding manually, i.e. without any support from AI, we kept human understanding and insight at the core of this key phase of the analysis which is predominantly concerned with gaining a deep understanding of the participant experience and the contextual information provided. Rather than relying on the support of a GPT to code the transcripts, we only introduced AI to our process after this stage at which point the codes, rather than participant quotes, were used as the unit of analysis for the framework analysis. Framework analysis relies, to a large part, on the recognition and consistent application of patterns across the dataset. Pattern recognition is an acknowledged strength of GPTs that are based on large language models (LLMs) (54) and by using the same set of refined prompts repeatedly, we were able to ensure a large degree of consistency in the mapping of all codes to the TFA and TDF.

Further research is likely needed to develop more robust and scalable methods in this area, but our study provides proof of concept that considered and robust use of generative AI is possible in qualitative research through built-in “human in the loop” processes that ensure quality control. Importantly, we did not ask the custom GPT to “do” the qualitative analysis, instead we worked with it, using it as a tool to stimulate our own thinking and interpretation of the data. In a way this is like working with human colleagues where coding of qualitative data is undertaken by one person and discussed and finalised with another. As yet, there are no recognised or validated methods for using generative AI in qualitative research, making the transparent sharing of any new approaches that teams are developing particularly important. Research in this area is ongoing and evolving rapidly, including the possible use of AI as a second coder for qualitative analysis (55), while still struggling to keep pace with the speed of development of AI, and also having to navigate the complexities of different generative AI models, subscription plans, institutional permissions, which all lead to differences in the functionality that is actually available to teams. Consequently, it may never be the case of there being one agreed upon method or technique.

### 4.4 Managing SMI and physical health

Despite ongoing efforts internationally, there is a growing body of evidence that suggests that the mortality gap experienced by people with SMI and driven by poor physical health is growing rather than decreasing (56). Large-scale RCTs of individual-level self-management interventions continuously show no long-term benefits to people with SMI across a range of outcome measures, including levels of physical activity (57), weight loss (58), blood cholesterol (59), and smoking cessation (60). Results from the DIAMONDS RCT of a diabetes self-management intervention for people with SMI are pending (61). These well-designed, robust, and highly-powered RCTs contribute important evidence to the notion that traditional self-management approaches do not achieve long-term lifestyle changes for people with SMI, even when they are based on rigorously co-designed, theory- and evidence-informed interventions.

In the diabetes literature, CGM is increasingly framed as a means for supporting behaviour change, rather than purely a blood glucose monitoring device (62–64), with positive impacts on self-management (65) and lifestyle changes (66). This evidence should be considered in the context of emerging findings that CGM can be successful for individuals with “poorly controlled” diabetes (67–69), with some studies highlighting the need for and impact of educational components (70), and support from a multi-disciplinary team that sit alongside the sensor itself (71). The need for more effective interventions and service pathways to support people with SMI to maintain good physical health is widely recognised and there is emerging evidence that CGM could play a role in this. Research in the general population has shown that structured CGM, i.e. the provision of CGM sensors alongside wrap-around education and support materials, is effective (11, 12), including for people who have previously struggled to manage their diabetes (72, 73). Future research will need to take into account identified implementation and equity challenges (74), especially if working in primary care (75–78) and/or with underserved populations (79–81).

Importantly, our study sends a signal that people with SMI are willing to engage with technology to manage their physical health, adding further weight to a growing body of evidence that challenges the outdated stigma that they cannot or do not want to do so (82). However, it is important to recognise that the context of a research study is not the same as the context of routine care, which may affect an individual’s willingness to try new care approaches (provided they are offered to them). In the case of this study, participants had already participated in the main part of the study, they had an existing relationship with the study team, and they were to some extent invested in the study, possibly paired with a degree of altruism that motivated them to take part in research to contribute to a greater good. While the concept of “therapeutic misconception” captures the notion that interventions received as part of a research study can feel inferior due to a need to stay within protocolised intervention parameters that may constrain what would normally be considered personalised or person-centred care (83), the research setting arguably offers a degree of information and support, as well as the explicit option to decline (by not giving consent), that is often lacking from routine care (84). Shared-decision making does often not take place in consultations with people with SMI (85), even though evidence suggests that the quality of diabetes care they receive is similar to that received by people without SMI (86, 87). The findings presented here should not be used to prompt the unsupported rollout of CGM to people with SMI, instead further research, co-produced with service-users, carers, and HCPs, is needed (88).

### 4.5 Limitations

We did not record details about the participants’ SMI diagnosis (type of SMI), the duration of their SMI diagnosis, or the duration of their diabetes diagnosis. This limits our ability to describe the sample in detail which, in turn, restricts the generalisability of our findings.

The SMI category encompasses conditions with meaningfully different symptom profiles, cognitive presentations, and pharmacological regimens. Schizophrenia spectrum disorders, schizoaffective disorder, bipolar disorder, and psychosis do not present or progress uniformly, and there are plausible reasons to expect that engagement with continuous monitoring may vary across these subgroups. For example, people with schizophrenia who experience paranoid ideation will likely have different perspectives and experiences of wearing a CGM compared with someone with bipolar disorder who experiences mood cycling. Furthermore, different medications have different metabolic side-effect profiles that will affect the resulting glucose data. In the absence of information about diagnostic subtypes in our dataset, our findings should therefore be interpreted as reflecting the acceptability of blinded CGM across the SMI population as a whole, rather than in relation to any single subgroup. Future studies with larger, purposively sampled cohorts have the potential to examine whether acceptability, engagement patterns, and support needs differ between subpopulations, so as to inform tailored implementation approaches.

The small sample size further limits the generalisability of our findings. It is important that this study and our findings are considered in the context within which they were conducted, i.e. as part of an exploratory test of feasibility and acceptability with the intention to provide preliminary insights that can serve as the foundation for future research. Participants had already engaged with a 16-week behaviour change intervention at the point of being asked to try CGM which might have contributed to their willingness to engage with the sensor and is not representative of people with SMI more generally. Participants’ motivation to try CGM was driven partially by a desire to support research. Therefore, assumptions should not be made that people’s willingness to try CGM in clinical practice would be the same. Furthermore, we only conducted interviews with people who wore the sensor. As such, our findings do not reflect the views of carers or of people who declined CGM. The perspectives of participants who wore the sensor but declined to take part in an interview might also have been different.

The blinded sensors we used in this study (FreeStyle Libre Pro) are licensed for research, but not clinical, use in the UK. As such, our study is not a real-life test of a technology that the participants are likely to have access to as part of the current or future diabetes care. Unblinded models are licensed in the UK for clinical use in both type 1 and type 2 diabetes (13, 89). We decided to investigate a blinded CGM system initially due to a lack of prior research in the field at the time. The decision to use a blinded system was made in collaboration with service-users and carers and was informed by our own experience of developing the “Change One Thing” app as part of the DIAMONDS intervention. Co-design of the intervention revealed that people with SMI were open to the idea of using a custom-built app and highlighted that additional support would be needed to ensure safe and successful use of any digital tools (29). For the study reported here, we did not have the means to design a bespoke app or put in place the necessary safety netting and support. At the time of the CGM wear period, we were aware that none of the participants had used the “Change One Thing” app in the DIAMONDS feasibility study. In the qualitative interviews that were undertaken subsequently, it became apparent that this was because of a general preference from participants not to use apps and instead work with a pen-and-paper intervention (90).

Specifically, we were keen to avoid the risk of overwhelm from an unblinded system leading to a deterioration in the participants’ mental health. Data overwhelm, and the general burden stemming from the complexity of the sensor, have been recognised as issues in the general population with diabetes (91). Our study did not provide information about how real-time feedback and interaction with a companion mobile app might affect acceptability and behaviour. As such, we present evidence here about the acceptability of wearing a sensor rather than of CGM used as part of diabetes self-management. Future research will need to explore acceptability and feasibility of unblinded systems, building on our findings.

A further limitation relates to design of the topic guide. Our semi-structured guide was primarily organised around acceptability of the sensor and the immediate experience of wearing it. While participants were encouraged to discuss their experiences freely our pre-specified focus on acceptability may have constrained the emergence of unanticipated themes that relate to broader issues around access to technological innovation, technology exclusion, and healthcare discrimination, all of which doubtless have implications for adoption of CGM in this population. Future qualitative work could adopt more open framings, and consider using complementary ad creative methods such as photo-elicitation interviews (92), capable of surfacing these dimensions of participants’ lived experience with health technologies.

### 4.6 Conclusions

By integrating mapping TFA and TDF mapping, we have produced a hybrid understanding about the connections between theoretical components of acceptability and modifiable determinants of behaviour. Our study offers a starting point for future intervention development by linking key drivers of acceptability (*Affective attitude* and *Burden*) to modifiable determinants of behaviour. TDF domains directly map to Mechanisms of Action which in turn link to specific Behaviour Change Techniques that can be operationalised in theory- and evidence-informed intervention development.

Integrated qualitative and quantitative findings from a small sample of people with SMI and type 2 diabetes suggest that CGM is feasible and acceptable to use with this population. Further research should address implementation and equity challenges, as well as wrap-around support needs. In the context of ongoing policy imperatives to reduce preventable mortality from metabolic conditions in people with SMI (, for example the NHS 10 year plan) (93), CGM could offer a complementary, low-burden approach to supporting engagement with physical health monitoring.

## Data Availability

The raw data supporting the conclusions of this article will be made available by the authors, without undue reservation.
