# Supplementary material for: Acceptability of blinded continuous glucose monitoring for people with serious mental illness and type 2 diabetes: findings from an exploratory mixed-methods study
Source: Front Clin Diabetes Healthc. 2026 Oct 7;7:1923186. doi: 10.3389/fcdhc.2026.1923186 (PMC13684867; doi:10.3389/fcdhc.2026.1923186)
Supplement: Supplementary file 1 [file DataSheet1.docx]

# Appendix 1 – Completed COREQ checklist

**Consolidated criteria for reporting qualitative studies (COREQ): 32-item checklist**

| **No. Item** | **Guide questions/description** | **Reported on Page #** |
| --- | --- | --- |
| **Domain 1: Research team and reﬂexivity** |  |  |
| *Personal Characteristics* |  |  |
| 1. Inter viewer/facilitator | Which author/s conducted the inter view or focus group? | 9 |
| 2. Credentials | What were the researcher’s credentials? E.g. PhD, MD | 9 |
| 3. Occupation | What was their occupation at the time of the study? | 9 |
| 4. Gender | Was the researcher male or female? | 9 |
| 5. Experience and training | What experience or training did the researcher have? | 9 |
| *Relationship with participants* |  |  |
| 6. Relationship established | Was a relationship established prior to study commencement? | **9** |
| 7. Participant knowledge of the interviewer | What did the participants know about the researcher? e.g. personal goals, reasons for doing the research | 9 |
| 8. Interviewer characteristics | What characteristics were reported about the inter viewer/facilitator? e.g. Bias, assumptions, reasons and interests in the research topic | 9 |

| **Domain 2: study design** |  |  |
| --- | --- | --- |
| *Theoretical framework* |  |  |
| 9. Methodological orientation and Theory | What methodological orientation was stated to underpin the study? e.g. grounded theory, discourse analysis, ethnography, phenomenology, content analysis | 10 |
| *Participant selection* |  |  |
| 10. Sampling | How were participants selected? e.g. purposive, convenience, consecutive, snowball | 8 |
| 11. Method of approach | How were participants approached? e.g. face-to-face, telephone, mail, email | 8 |
| 12. Sample size | How many participants were in the study? | 14 |
| 13. Non-participation | How many people refused to participate or dropped out? Reasons? | 14 |
| *Setting* |  |  |
| 14. Setting of data collection | Where was the data collected? e.g. home, clinic, workplace | 14 |
| 15. Presence of non-participants | Was anyone else present besides the participants and researchers? | 14 |
| 16. Description of sample | What are the important characteristics of the sample? e.g. demographic data, date | 12 |
| *Data collection* |  |  |
| 17. Interview guide | Were questions, prompts, guides provided by the authors? Was it pilot tested? | Supplement |
| 18. Repeat interviews | Were repeat inter views carried out? If yes, how many? | **N/A** |
| 19. Audio/visual recording | Did the research use audio or visual recording to collect the data? | 9 |
| 20. Field notes | Were ﬁeld notes made during and/or after the inter view or focus group? | 14 |
| 21. Duration | What was the duration of the inter views or focus group? | 14 |
| 22. Data saturation | Was data saturation discussed? | N/A |
| 23. Transcripts returned | Were transcripts returned to participants for comment and/or correction? | N/A |
| **Domain 3: analysis and ﬁndings** |  |  |
| *Data analysis* |  |  |
| 24. Number of data coders | How many data coders coded the data? | 10 |
| 25. Description of the coding tree | Did authors provide a description of the coding tree? | Table 2 |
| 26. Derivation of themes | Were themes identiﬁed in advance or derived from the data? | Page 14 onwards |
| 27. Software | What software, if applicable, was used to manage the data? | **N/A** |
| 28. Participant checking | Did participants provide feedback on the ﬁndings? | **N/A** |
| *Reporting* |  |  |
| 29. Quotations presented | Were participant quotations presented to illustrate the themes/ﬁndings? Was each quotation identiﬁed? e.g. participant number | Results section |
| 30. Data and ﬁndings consistent | Was there consistency between the data presented and the ﬁndings? | **Results section** |
| 31. Clarity of major themes | Were major themes clearly presented in the ﬁndings? | **Results section** |
| 32. Clarity of minor themes | Is there a description of diverse cases or discussion of minor themes? | **N/A** |

# Appendix 2 – Participant information pack

[Researcher’s contact details]

[Name and address of participant]

[Date]

Dear [first name],

**Invitation to give feedback about using technology to support your health**

Thank you for taking part in the DIAMONDS study! Your time and support are much appreciated.

On [*date*] you had an appointment where you were offered a continuous glucose monitor, called Freestyle Libre. I understand that you [*agreed/declined*] to wear this.

[*Thank you for agreeing to wear the monitor. This is the first time these have been offered to people with a severe mental illness as part of a research study. I am hoping to learn more about what it is like to wear such a device and how technology can help you to look after your health and wellbeing.*

*To find out as much as we can about this important topic, I would like to invite you to take part in an interview where you can tell me more about what it was like to wear the monitor. I will also ask you to fill out two short questionnaires about your experience using the device. And I am interested to find out more about other technology, such as step counters or apps, that you might use to support your health and wellbeing.*

*/*

*Thank you for considering to wear the monitor. It is absolutely not a problem that you didn’t want to wear it. Your care will not be affected.*

*I would like to understand more about if and how you use technology, such as step counters or apps, to support your health and wellbeing. To do this, I would like to invite you to an interview where you can tell me more about the different ways you manage your health.*]

I have enclosed a Participant Information Sheet which gives more information. I’m also sending you a Consent Form to take part in the interview.

I will call you in the next couple of days to see if you would like to take part. I will be happy to answer any questions you may have at that point.

[*I have also enclosed some information about these interviews for a family member or friend who acts as a carer for you. If you have a family member or friend who cares for you, I would be very grateful if you could pass this information to them.*]

I look forward to speaking to you soon, but if you have any questions in the meantime, please contact me using the details at the top of the page.

Thank you!

Yours sincerely,

[Researcher’s name]

[Study logo]

Participant Information Sheet – Feedback sessions about technology to support and health and wellbeing

Thank you for taking part in the DIAMONDS study, your contribution to the research is much appreciated.

At this point in the study, I would like to invite you to an interview to find out more about the ways you use technology, such as step counters or apps, to support your health and wellbeing. [*I would also like to speak to you about what it was like to wear the blood sugar sensor on your arm.*]

Before you decide, please take time to read the following information carefully.

- [*You are being asked to share what it was like wearing the blood sugar sensor (continuous glucose monitor) on your arm. You will be asked to fill in two short questionnaires as well as a number of questions during the interview.*]
- You are being invited to talk about the different ways (if any) you use technology to support your health and wellbeing.
- This will involve a one-off appointment with a researcher lasting approximately 60 minutes (1 hour).
- We will agree together how and where the interview will take place. This could be in your home, at a community venue, over the phone, or as a video call.
- If you decide to take part, you will be asked to complete a consent form (enclosed). A consent form is a written record that you’re happy to take part in the interview.
- With your permission, I will audio record the interview. When I report the findings of the research, I will not use your name or any information which could identify you.
- If you would prefer me not to record the session, I will take detailed notes of your responses instead.
- You do not have to take part and you can stop taking part at any time without giving a reason. Your decision about whether or not to take part will not impact the care you receive.
- **You do not need to do anything at this stage. I will contact you in the next few days to check if you would like to go ahead with the interview. If you do, we will agree where and when we will meet.**
- **I will support you to complete the consent form before the start of the interview.**

How to get in touch	Contents

If you have any questions about the study, you can contact me: Jennifer Brown, DIAMONDS Programme Manager:

Tel:	01904 321662

Mobile:	07384 239004

Email:	[jennifer.brown@york.ac.uk](mailto:jennifer.brown@york.ac.uk)

There is an answering machine available 24 hours a day, so please leave a message and I will return your call as soon as possible.

You can find out more about the DIAMONDS study on our website: [www.diamondscollaboration.org.uk](http://www.diamondscollaboration.org.uk)

1 Why am I being asked to take part?
2 What will taking part involve?
3 Do I have to take part?
4 Possible benefits and disadvantages of taking part
5 Will the information I provide be confidential?
6 More information about taking part
7 What do I need to do now?
9 What do I need to do now?

Why am I being asked to take part?

1

One aim of the DIAMONDS study is to find out more about how people with severe mental illness and diabetes use technology, such as step counters or apps, to support their health and wellbeing.

Technology is being used more and more in health care. It is important to understand if and how people with severe mental illness engage with the these offers. This is to ensure that everyone receives the support they need, and nobody misses out.

[*In particular, I would like to know about what it was like to wear the continuous glucose monitor (Freestyle Libre). This is the first time these devices have been offered to people with a severe mental illness as part of a research study.*

*I would like to find out about what it was like for you to wear the monitor, if you had any problems with it, and if you could imagine wearing one again in the future.*

*In future studies, these devices might be offered to a larger number of people, and it is important for us to understand your experience first.*]

[*If you have a friend or family member who provides informal care to you then we are interested in talking to them too. We can do this separately or at the same time*.]

What will taking part involve?

2

**What happens next?**

When I contact you again in the next couple of days, you will have a chance to ask any questions you have about taking part.

I will then ask you if you would like to take part in an interview.

If you confirm that you do want to take part, we will agree an appointment and how and where the interview will take place.

If you do not want to take part, you will not be expected to do anything further for this part of the study. You may still be contacted again by the DIAMONDS team for other reasons.

**At the appointment**

At the start of the appointment, we will go through the consent form together. This will need to be completed as a formal record that you’re happy to take part. We will either do this on paper, where you will be able to complete and sign the form yourself, or over the phone, where I will record your answers and sign the form on your behalf.

If you change your mind before our appointment, you will not need to complete a consent form and you will not need to take part.

In total, the appointment will not take longer than approximately 60 minutes (1 hour). It will be relaxed and informal, with no right or wrong answers.

The interview can take place at your home, a community venue, over the phone, or as a video call. We will decide together what will be best for your interview.

We can reimburse childcare and travel expenses that you incur in taking part. If you prefer, you are welcome to have a family member, friend, or carer with you during the interview.

**What will I be asked about?**

The interview will be about your experience of using technology to support your health and wellbeing. The questions will be about any apps or devices you might use to manage your health. These could be on your phone or tablet or on a separate device. I will be interested to know how you feel about using technology, if your doctors have offered you any technology for your health, and if you feel you’ve been given the right support to use technology in this way.

[*I will also ask you about the glucose monitor (Freestyle Libre). This will include questions about having it fitted, wearing it day-to-day for two weeks, and removing it, as well as any problems you might have had with the device. During the appointment, I will also ask you to fill in two short questionnaires about the continuous glucose monitor.*]

Do I have to take part?

3

You do not have to take part. You can change your mind about taking part at any point.

If you do take part, you are free to stop at any time without giving a reason. Your decision will not affect any care you receive.

If you decide to stop during the interview, we will discuss if you are happy for me to use the responses you have already given.

Benefits and disadvantages of taking part

4

What are the possible benefits of taking part?

I cannot promise that taking part will benefit you. However, you will make an important contribution to the research by helping me understand how you use technology to manage your health [*and what it was like to wear the glucose monitor*].

[*You will be helping us to learn more about what support people with severe mental illness and diabetes might need to get the most out of wearing a continuous glucose monitor.*] Your feedback will inform future research studies in this area which will contribute to better care for people with severe mental illness and diabetes

What are the possible disadvantages of taking part?

Taking part will take up some of your time. I will arrange to talk to you at a day, time and location that is most convenient for you.

Will the information I provide be confidential?

5

Your involvement in this part of the study will be confidential and only members of the research team will know whether or not you have agreed to take part. We will not inform your GP or any other healthcare providers about whether or not you have taken part.

The interview will be digitally audio recorded (with your permission). We will transcribe the recording verbatim. This means that we will write down what you have said exactly as you said it. The recording will be stored securely for a period of 10 years after the study is finished; then it will be destroyed.

If you don’t want your interview to be recorded, any notes I have taken of your responses will also be stored securely for a period of 10 years after the study is finished; then they will be destroyed.

I hope to present findings of the research in scientific journals or at conferences, as well as on the DIAMONDS website and social media. I will not include your name or any information that could identify you.

6

More information about taking part

What will happen to information collected about me during the study?

I will treat any information you give me in confidence. I will store all your information safely and securely. I will not mention your name in any publications about the study and I will make sure that no individuals can be identified in the study results.

Sometimes researchers are asked to share information we collect in studies with colleagues who are looking at similar ways to improve healthcare. With your agreement, I would like to be able to share the information I collect about you as part of this study. I will always remove all identifiable information before I share your information. This is controlled by strict University of York and NHS Trust procedures.

I will keep the information you give me as part of the current study safely for 10 years after the study ends. It will then be destroyed securely. Paper records will be handled only by the study team and kept in locked cabinets. Electronic records will be held on a secure computer server approved by the University of York. Only authorised members of the study team will have access to your information.

I would be happy to answer any questions you may have about how I use the information I receive from you as part of the study.

What if there is a problem?

If you have concerns about the study or if there is anything you would like to ask about the study, please get in touch. My contact details are on page 1. If you would prefer you may contact the Chief Investigator: Prof Najma Siddiqi, Tel 01904 321681, email: najma.siddiqi@york.ac.uk.

It is not anticipated that any harm will to come to you from being in this study. However, if you wish to complain or have any concerns about any aspect of the way you have been approached or treated during the course of the study, the normal NHS complaints processes are available to you through your local Patient Advice and Liaison Service (PALS) <insert PALS number>.

If you wish to raise a complaint on how we have handled your personal data, you can contact the University of York’s Data Protection Officer who will investigate the matter (dataprotection@york.ac.uk). If you are not satisfied with our response or believe we are processing your personal data in a way that is not lawful you can complain to the Information Commissioner’s Office (ICO) whose details can be found here: https://ico.org.uk/.

What will happen to the results of the study?

When the study has finished and the results have been published, I will send you a summary of the study results if you have agreed for me to do so. You can contact me directly if you decide not to take part in the study but would still like to receive a summary of the results. I will also keep the study website up to date with news of the study.

Who is organising and funding the study?

This study is organised by Bradford District Care NHS Foundation Trust and a group of Universities, including the universities of York, Leeds, and Southampton. This group is working with NHS Trusts and GP practices in England.

The study has been funded by the Department of Health’s National Institute for Health Research Programme Grants for Applied Research programme. The study has been reviewed by an independent group of people, called a Research Ethics Committee. This is to protect your safety, rights, wellbeing, and dignity. This study was looked at and approved by the Leeds West Research Ethics Committee (REC Reference 21/YH0059).

What do I need to do now?

7

Right now, you don’t need to do anything. You may want to discuss the information in this letter with a family member or friend. If you want, you can have a read of the enclosed consent form.

[*If you have anyone who provides informal care to you, such as a family member or friend, please pass the carer information pack to them.*]

I will contact you in a couple of days. You will have the chance to ask any questions you have about taking part. If you’re happy to go ahead, we will agree an appointment for your interview.

Thank you for taking the time to consider taking part in this study.

Data Protection statement

The University of York is the sponsor for this study based in the United Kingdom. We will be using information from you in order to undertake this study and will act as the data controller for this study. This means that we are responsible for looking after your information and using it properly.

The University of York is a publicly funded organisation that conducts research to improve health, care and services. Research following the UK Policy Framework for Health and Social Care Research is conducted to serve the interests of society as a whole. This means that the University of York is using the legal basis provided under the Data Protection Act 2018 and the General Data Protection Regulation of ‘for the purposes of research conducted in the public interest’ to use your personal data for this research.

Your rights to access, change or move your information are limited, as we need to manage your information in specific ways in order for the research to be reliable and accurate. If you withdraw from the study, we will keep the information about you that we have already obtained. To safeguard your rights, we will use the minimum personally-identifiable information possible.

You can find out more about how we use your information at:

<https://www.york.ac.uk/healthsciences/research/trials/trials-gdpr/>

<https://www.york.ac.uk/healthsciences/research/trials/trials-gdpr/research-partcipants/>

Participating NHS bodies and The University of York will collect information from you for this research study in accordance with the sponsor’s instructions.

The University of York will use your name and contact details to contact you about the research study, and make sure that relevant information about the study is recorded for your care, and to oversee the quality of the study. The University of York will pass these details to the participating trust along with the information collected from you. The only people in the participating trust who will have access to information that identifies you will be people who need to contact you about the research you are taking part in, to invite you to take part in other research studies you may be interested in or audit the data collection process. The people who analyse the information will not be able to identify you and will not be able to find out your name or contact details. Individuals from University of York and regulatory organisations may look at your medical and research records to check the accuracy of the research study.

The University of York will keep consent forms and other information about you from this study for 10 years after the study has finish. Contact details will be kept for three years unless you have given consent to be contacted about future research.

Thank you for reading this information.

[Study logo]

**PARTICIPANT CONSENT FORM – Interviews about technology to support health and wellbeing**

**Title of Study: DIAMONDS**

**IRAS ID: 279019**

**REC Reference: Leeds West REC 21/YH/0059**

**Study ID:**

If you wish to take part in an interview about your experience of using technology to support your health and wellbeing, **please place your initials in each of the boxes below, sign and date this form.**

*Please* ***initial***

*each box*

***Initials***

***Initials***

***Initials***

***Initials***

***Initials***

***Initials***

1. I confirm that I have read and understand the information sheet version [*XX*] dated [*XX*] for this part of the study and that I have had the opportunity to ask any questions about the study.
2. My questions have been answered to my satisfaction.

3. I understand that my participation is voluntary and that I am free to stop at any time without giving any reason, and without my medical care or legal rights being affected.
4. I agree to the University of York holding copies of my consent form, other study related documents and my contact details to allow them to contact me for appointments and send me letters and questionnaires.
5. I understand that the information collected about me could be used to support other research in the future, and may be shared anonymously with other researchers
6. **I agree to take part** **in the interview.**

| In addition to the above statements please initial the following boxes to indicate whether you agree with the following statements. Your participation in this research study will not be affected if you do not agree with these statements.  **Yes No**  ***Initials***  ***Initials***  I am happy for the interview to be audio recorded.  ***Initials***  ***Initials***  I am happy to be contacted about future DIAMONDS studies.  I would like to receive a summary of the study findings.  ***Initials***  ***Initials*** | | | | | | | | | | | | | | | | | | | | | | | | | | |  |
| --- | --- | --- | --- | --- | --- | --- | --- | --- | --- | --- | --- | --- | --- | --- | --- | --- | --- | --- | --- | --- | --- | --- | --- | --- | --- | --- | --- |
|  | |  | *d* | | *d* | | */* | | *m* | | *m* | | */* | | *y* | | *y* | | *y* | | *y* | |  | |  |  |  |
| Print name |  | | |  | |  | | / |  |  | | / | | 2 | | 0 | |  | |  | |  | | Signature | | | |
| Name of participant (*please print*) | | | | Date | | | | | | | | | | | | | | | | | | Signature of participant | | | | | |
|  | | | | *d* | | *d* | | */* | *m* | *m* | | */* | | *y* | | *y* | | *y* | | *y* | |  | | | | | |
| Print name |  | | |  | |  | | / |  |  | | / | | 2 | | 0 | |  | |  | |  | | Signature | | | |
| Name of person taking consent  (*please print*) | | | | Date | | | | | | | | | | | | | | | | | | Signature of person taking consent | | | | | |

**[One copy to be kept in Trial Master File; one copy given to participant]**

# Appendix 3 – Interview topic guides

Attitudes towards technology-supported self-management and user experience of continuous glucose monitoring

Topic guides for semi-structured qualitative interviews

## Service users

*Service users, i.e. individuals living with type 2 diabetes and a severe mental illness who are participants in the DIAMONDS feasibility study, will be interviewed about their attitudes towards and experience of using technology to support their self-management.*

*Where appropriate, the second part of the interview will explore the experience of wearing the Freestyle Libre Pro continuous glucose monitor.*

*“Completers” will be defined as those participants who agreed to wear the CGM and continued to wear it for at least 10 days of the 14-day wear period.*

*“Non-completers” will be defined as those participants who agreed to wear the CGM and wore it for less than 10 days (for whatever reason).*

*“Non-wearers” will be defined as those participants who declined to wear the CGM/did not wear the CGM.*

### Part 1 – Interview topic guide for all service users

Questions will address the following themes:

1. Self-management routines, incl. medications, diet, appointments, routines, exercise/activity, social events/contacts
2. Experience of using technology (apps or devices) to support self-management
3. Use of technology to support diabetes self-management

### Part 2 – Additional themes for completers and non-completers only

Questions for completers and non-completers (see above) will address the following additional topics:

1. Reasons for agreeing to wear the CGM
2. Feelings (positive, curious, anxious, nervous etc.) prior to having the CGM fitted compared with feelings now
3. Experience (likes, dislikes, problems) of wearing the CGM (incl. fitting and removal)
4. Thoughts about wearing a CGM again in the future and/or wearing a different (unblinded) model

### Part 2 – Additional themes for non-wearers only

Questions for non-wearers (see above) will address the following additional topics:

1. Reasons for not wearing the CGM (if the participant is happy to disclose this)
2. Thoughts about wearing a CGM in the future and/or wearing a different (unblinded) model

## Carers/family members

*Informal carers, i.e. spouses, other family members or friends providing unpaid support with activities of daily living, will be interviewed about their experience of supporting the service user in using technology to support their health and wellbeing. Questions will also include the experience of the service user wearing the CGM.*

*Carers of completers and non-completers, as defined above, will be invited to take part in an interview.*

*Carers of non-wearers will not be approached for an interview.*

### Interview topic guide for all carers

Questions will address the following themes:

1. Experience of being an informal carer
2. Experience of offering support for using technology (apps or devices) to support self-management
3. Experience of offering support during CGM wear
4. Thoughts about repeated CGM wear in the future and/or wear of an unblinded model

# Appendix 4 – Gem instructions

**Description**: Your task is to support me with a qualitative framework analysis based on the TFA and TDF.

**Instructions**: You are an academic analysis tool to support a specific phase of a multi-stage qualitative analysis in the context of a research project in health sciences. The focus of the work is on the acceptability of continuous glucose monitoring (CGM) to people who have serious mental illness and type 2 diabetes.

The basis of the analysis are nine transcripts from qualitative interviews that explored participant's experience of wearing a CGM sensor in the context of their lives with SMI. These transcripts have been inductively coded and organised into four summary themes. **A coding matrix has been developed and will be uploaded by the user as a .csv file.**

When asked to access data from the spreadsheet, you must ensure 100% accuracy at all times, using quotes *exactly* as they are recorded in the spreadsheet, without paraphrasing.

The aim for this phase of the analysis is to map the codes from the spreadsheet to the domains of the Theoretical Framework of Acceptability (TFA) and then to the domains of the Theoretical Domains Framework (TDF).

I have added a PDF called "Sekhon TFA 2017" to your knowledge base. This is to be your main source of information for mapping of codes against the TFA. If you use any other sources you *must* flag this clearly in your response.

I have also added two PDFs as your knowledge base about the TDF: "Cane TDF 2012" and "Atkins TDF 2017". Use these as your main source of knowledge about the TDF. If you use other sources, you *must* make me aware of this.

You *must* use the Python interpreter to pull raw data for every single code and quote from any uploaded file before providing a response.

**1. Data Input Structure (Ground Truth)**

The Unit of Analysis: You must treat the Code as the primary unit of analysis.

Vertical Alignment: You will process every row as an atomic record containing: Code Number, Code Name, Theme Name, Subtheme (or "--"), Participant ID, and Quote.

Mapping Hierarchy: You must maintain the inductive context (Theme and Subtheme) as provided in the spreadsheet while performing the deductive mapping.

Non-Negotiable Allocation: You will use the quotes exactly as they are assigned to the codes in the spreadsheet. You must not re-code or suggest alternative placements.

**2. Theoretical Frameworks (Main Sources)**

TFA (Theoretical Framework of Acceptability): You must use Sekhon (2017) as the primary source for mapping and definitions (e.g., Affective Attitude, Burden, Ethicality, Intervention Coherence, Opportunity Costs, Perceived Effectiveness, Self-efficacy).

TDF (Theoretical Domains Framework): You must use Cane (2012) and Atkins (2017) as the primary sources for mapping and definitions (e.g., Knowledge, Skills, Environmental Context and Resources, Beliefs about Consequences, etc.).

**External Sources: If you use any other source, you must flag this clearly in the response.**

**3. Analytical & Mapping Principles**

Interpretation Column: You must explicitly explain why the mapping exists by linking the TFA construct to the TDF construct (e.g., how a specific "Burden" in TFA relates to "Environmental Context and Resources" in TDF).

Honest Gaps: If a mapping feels forced rather than a good fit, you must leave a gap. Do not force data into a construct where it does not naturally belong.

Conflicting Data: If a code contains both positive and negative experiences, you must include one representative quote for each to show the full range.

No Imputation: You must never fabricate or "fill in" missing data. Treat a dash (--) as an intentional signal that no subtheme exists.

**4. Output Format (Markdown Table)**

You will always use this specific five-column structure:

Code - Code Number & Name

Key Illustrative Quote (participant ID) - Exact quote from CSV (ID)

TFA Domain(s) - Mapped Construct(s)

TDF Domain(s) - Mapped Domain(s)

Interpretation - Explanation of the TFA-TDF link

**5. Quote Integrity**

Zero Paraphrasing: You must ensure 100% accuracy, using quotes exactly as they are recorded in the spreadsheet. Do not add embellishments.

Selection: You will select the single most representative quote for the code, unless conflicting data requires two quotes to show a range.

**I. The Iterative Workflow (The "Code-by-Code" Rule)**

To ensure 100% alignment and minimize "token burn," the analysis must proceed one code at a time using this three-step sequence:

The Menu & Filter: You will provide a "Quote Menu" (all quotes for the code) and a "TFA Filter" (preliminary assessment of whether the code is Intervention-specific or Contextual/Gap).

User Curation: The user will select the specific quotes to be used and confirm/adjust the TFA status.

The Final Mapping: You will generate the single-row Markdown table based on your selection.

**II. Theoretical Guardrails**

TFA Applicability: You must only map to the Theoretical Framework of Acceptability (TFA) if the participant is evaluating or responding to the CGM intervention specifically.

The Contextual Gap: General lifestyle habits, existing self-management strategies (e.g., diet/exercise), or SMI symptoms must be marked as a TFA Gap, as they describe life context rather than intervention acceptability.

TDF Specificity: Mapping to the Theoretical Domains Framework (TDF) should use specific construct language (e.g., "selective focus" for Memory, Attention and Decision Processes or "mental representations of outcomes" for Goals).

**III. Formatting & Style Standards**

Table Structure: Maintain the five-column Markdown structure (Code, Quote, TFA, TDF, Interpretation).

Quote Integrity: Use quotes exactly as they appear in the CSV. Use bold Participant IDs (e.g., 300100) within the quote cell to separate multiple entries.

Interpretation Style:

Conciseness: Keep the analysis brief and direct.

No Redundancy: Do not repeat Participant IDs or the code name within the Interpretation text.

The Link: Explicitly explain the relationship between the TFA domain (if applicable) and the TDF domain.

As we work through this analysis together, I will provide feedback on your outputs and will ask you to make revisions where this is necessary.

# Appendix 5 – Coding matric table

| **Code** | **Illustrative quote(s) (participant ID)** | **TFA Domain(s)** | **TDF Domain(s)** | **Interpretation** |
| --- | --- | --- | --- | --- |
| ***Theme 1: The context of living with SMI and physical health conditions*** | | | | |
| **12 Medication adherence** | "I don't have any problems taking them [medicines]." (300 100)  "I was on Metformin but you take one in the morning, one at night and I always forgot to take my morning one." (500 104)  "But one week I've missed five morning doses, that's ten tablets of Metformin. And then the other weeks I've averaged about two missed doses a week, so that's four tablet a week missed." (200 105) | -  *Description of current behaviours, not CGM acceptability.* | -  *Description of general clinical context, not CGM-specific determinants of behaviour.* | - |
| **13 Diabetes is an invisible illness** | "With the diabetes nurse you see them twice a year so automatically your head is telling you, well it is not obviously as important." (500 104)  "...there’s no real purpose for taking the morning dose, you see, my evening tablets send me to sleep and I can tell when they’ve worn off and I need some more. But because the morning tablets are just Canagliflozin, and Metformin, there’s nothing that’s, you know, I don’t think this is due, there’s nothing, there’s no precursor to say that it’s due, you know?" (200 105) | -  *Description of current behaviours, not CGM acceptability.* | **Knowledge  Beliefs about Consequences** | The "invisibility" of diabetes relates to Knowledge (awareness of the asymptomatic nature of diabetes) and Beliefs about Consequences (the absence of a physical "precursor" or immediate symptom leads to a belief that medication/monitoring has "no real purpose"). |
| **17 Managing appointments** | "I’m on clozapine so I go in every four weeks for a blood test. And then... I had the whatsit things, diabetes check blood tests as well" (300 100)  "if I’ve got any appointments, I make sure I get there early. I always like to be at appointments really early." (400 103) | -  *Description of the baseline clinical burden of managing appointments, not CGM acceptability.* | **Environmental Context and Resources  Memory, Attention, and Decision Processes** | The appointment schedule constitutes the Environmental Context and Resources that frame the participant's routine. Navigating this requires significant cognitive work, specifically Memory (the effort to remember appointments) and proactive Decision Processes (choosing to arrive "really early). |
| **19 Relationship strain due to SMI** | "[Partner's name] doesn’t… my symptoms frighten him and he really struggles to talk about it." (500 104)  "My brother’s in [city], so I have a family but […] he doesn’t talk to me, he only knew me in my teens when I was really, really poorly with it all.  And we couldn’t see each other for a long time and I’m 53 now so we haven’t seen each other for a long time." (200 105) | -  *Reflection of social context, not CGM acceptability.* | -  *Description of social context, not CGM-specific determinants of behaviour.* | - |
| **26 Impact of overweight/obesity** | "I can't do star jumps because I am too big, I can't jump. I can't do press ups" (500 104)  "When I was put on Clozaril they did say it can come with weight gain. After a few years I had just put on that much weight that quickly that I developed the diabetes type 2." (500 104) | -  *Description of obesity as a contextual factor, not CGM acceptability.* | -  *Description of obesity as a contextual factor, not CGM-specific determinants of behaviour.* | - |
| **46 SMI dominates** | "Diabetes is easy to manage but schizophrenia is worst thing in my life." (400 102)   "I never thought about my diabetes before really because I was that concerned about the schizophrenia and the hallucinations... [...] I always thought my mental health was the more important thing." (500 104) | -  *Description of context, not CGM acceptability.* | Social/Professional Role and Identity | Participants describe a hierarchy of clinical concern where the management of schizophrenia is prioritised over diabetes care. This prioritisation is driven by the participant's primary identity and role as a mental health service user. |
| **48 Experience of healthcare** | "...the doctors don’t seem to care, really, you know. […] Some doctors don’t even know anything about mental illness, you know what I mean? They've heard about it and read about it, but really deep down, they don’t know what a person goes through every day with paranoia or mental illness. […] if you’re not actually feeling what you’re going through and living it day-in, day-out, they haven’t got a clue." (400 103)  "She [nurse] said, I will get back in touch with you around March time. She never did." (500 104) | -  *Describes interactions with healthcare, not CGM acceptability.* | **Social Influences  Environmental Context and Resources  Social/Professional Role and Identity** | Experience of missed follow-ups maps to Environmental Context and Resources, representing a systemic barrier. The perception of a lack of empathy and understanding of SMI maps to Social Influences and Social/Professional Role and Identity, highlighting a perceived disconnect between professional medical knowledge and the patient's lived experience. |
| **50 Impact of SMI symptoms/medication** | "If I was really tired and I couldn’t sleep I would start getting worked up and then my hallucinations got a lot worse." (500 104)   "My mental health affects me every single day of my life. I'm never in peace, hardly. I have restlessness. […] I get paranoid with people…" (400 103) | -  *Description of a contextual burden, not CGM acceptability.* | -  *Description of a contextual burden, not CGM-specific determinants of behaviour.* | - |
|  | | | | |
| ***Theme 2: Existing self-management strategies*** | | | | |
| **15 Use of available financial resources** | “...when I factor it all out I’m left with £250 to actually pay the bills, run a car and eat. Basically it’s just not realistic.” (200 105) | -  *Contextual, not CGM acceptability* | **Environmental Context and Resources  Optimism** | Maps to Environmental Context and Resources through the lack of material resources and the stress of living costs. Reflects limited Optimism, negative appraisal of costs that are "just not realistic." |
| **16 Motivation to make positive lifestyle changes** | "Before I know it I am doing squats every time I am washing up and then every day because I love music I put my favourite song on and dance to it…" (500 104)   "I know I can do better and I will do better. It's just going to take time, that's all." (400 103)   "Losing weight. Yeah, I want to get on that bus and go to see my daughter, I've got a granddaughter [...], she's beautiful." (300 103) | -  *Motivation/intentions, not CGM acceptability* | **Goals  Intentions  Behavioural Regulation  Optimism  Beliefs about Capabilities** | Goals and Intentions map to the "mental representations of outcomes" and "conscious decisions" to act. Behavioural Regulation is seen in the management of daily activity habits (e.g., squats while washing). Optimism and Beliefs about Capabilities reflect the participants' "confidence that things will happen for the best" and their "perceived competence" regarding self-improvement. |
| **18 Support from family** | "But I can't do anything myself. Once my mum gets me or whatever, then we can walk for absolutely miles but I can't leave the house on my own." (500 104)   "So I’ve no family, my friends are, old friends, going back, so I’d have been 15 years old, and we just keep in touch on Facebook." (200 105) | -  *Contextual, not CGM acceptability* | **Social Influences  Environmental Context and Resources  Beliefs about Capabilities** | Maps to Social Influences through the "interpersonal processes" of family support, influencing health behaviours. Environmental Context and Resources is identified where a family member serves as a facilitator. Beliefs about Capabilities is reflected in the dependency on others that limits self-confidence to manage independently. |
| **20 Support from friends** | "… sometimes I have a friend who sort of stays over … having that company really helps me because I feel safe when I've got that company." (400 103)   "So I've no family, no friends, nobody that helps me, no." (200 105) | -  *Contextual, not CGM acceptability* | **Social Influences  Environmental Context and Resources  Emotions** | Maps to Social Influences (interpersonal processes and social support underpin health tasks), Environmental Context and Resources (presence/absence of friends as a situational facilitator), and Emotions (social proximity as a regulator of psychological states like "safety"). |
| **21 Experience using SMBG** | "It doesn’t hurt or anything, it is not that side of it. It is getting everything out and getting the instruction booklet out." (500 104)   "Interviewer: How do you find that [checking your blood sugar]? Respondent: It's very easy." (400 102) | -  *Contextual, not CGM acceptability* | **Environmental Context and Resources  Skills  Beliefs about Capabilities  Emotions** | Maps to Environmental Context and Resources regarding testing burden and requirements. Skills and Beliefs about Capabilities reflect the spectrum of testing proficiency. Emotions is linked to the participant's appraisal of the task based on the absence of negative "physical sensations" like pain. |
| **28 Successful lifestyle changes** | "I managed to get my weight down to something like 14 stone seven. It is a few stone lighter than what I was a few years ago but it is still a lot heavier than the first 20 odd years of my life." (500 104) | -  *Contextual, not CGM acceptability* | **Beliefs about Capabilities** | Maps to Beliefs about Capabilities because past success in weight management contributes to "perceived competence" and "self-confidence" regarding the individual's ability to influence their health outcomes. |
| **29 Support from professional carers/HCPs** | "So she [nurse] showed me and I am like, no, I can't do that. They [exercises] looked different than that." (500 104)   "My CPN is trying to organise so I can go swimming." (100 102) | -  *Contextual, not CGM acceptability* | **Social Influences  Environmental Context and Resources  Beliefs about Capabilities** | Social Influences are demonstrated through professional "supervision" and "social support". The CPN functions as an "organisational" resource within Environmental Context and Resources. Attempt to teach exercises directly impacts the participant's Beliefs about Capabilities, specifically their "perceived competence". |
| **30 Social contacts** | "I was walking with all my friends." (500 104)   "I'm constantly in touch with people on my phone" (400 103) | -  *Contextual, not CGM acceptability* | **Social Influences  Environmental Context and Resources** | Maps to Social Influences through "interpersonal processes" of social support and maintaining friendships. Relates to Environmental Context and Resources as the "phone" serves as a material resource used to facilitate social interactions. |
| **31 Use of health tech** | "No, my partner has set it up with my fitbit to... I don’t use it, I really don’t." (500 104)   "I used to have a step counter, but I don’t know where that went to. I'd love to put a step counter onto my phone again. I’ll ask the support worker when he comes today." (400 103) | -  *Contextual, not CGM acceptability* | **Skills  Environmental Context and Resources  Social Influences  Intentions** | Skills is relevant as participants often find technology difficult to navigate. Environmental Context and Resources and Social Influences are linked through the reliance on "partners" and "support workers" to facilitate access to or setup of technology. Intentions are demonstrated by the conscious decision and "resolve to act" regarding re-installing a step counter. |
| **32 Aware of the importance of diet** | "I've not got a very good diet. My diet's atrocious." (400 103)  "I watch what I eat. […] I've cut down on my sugar." (300 103) | -  *Contextual, not CGM acceptability* | **Knowledge  Behavioural Regulation** | Reflect Knowledge regarding health rationales and the use of Behavioural Regulation (self-monitoring and breaking habits) to manage intake. |
| **37 Aware of the importance of physical activity** | "I walk every day for an hour or more. [...] And I also work at a zoo once a week at the moment, so that keeps me fit." (300 100)  "I am like, I thought in my head, right, there is a list of different exercises I can do in the house, sit ups, stuff like that. But I never did because I was always like, I will just wash up and then I will do it. I will just clean that then I will do it. I just get washing in and then I will do it." (500 104) | -  *Contextual, not CGM acceptability* | **Knowledge  Intentions  Memory, Attention and Decision Processes  Behavioural Regulation** | Captures the range of physical activity engagement: from established Behavioural Regulation to Knowledge and Intentions that are disrupted by domestic tasks competing for Memory, Attention and Decision Processes (specifically selective focus). |
| **38 Current glucose monitoring** | "I had had a kit for about 12 months, but I just didn’t really use it unless I felt really unwell." (500 104)   "I don’t have to [check blood glucose], no, I don’t have to." (200 105)   "I try, I check it [blood sugar] every day, twice." (400 102) | -  *Contextual, not CGM acceptability* | **Behavioural Regulation  Memory, Attention and Decision Processes  Knowledge  Skills** | Maps to Behavioural Regulation through the presence or absence of a monitoring routine. Memory, Attention and Decision Processes is demonstrated using internal physical cues (feeling unwell) as a "decision" trigger for testing. Knowledge is reflected in the participant’s understanding of their clinical requirements ("I don't have to"), while the ability to test daily implies the necessary Skills to operate traditional equipment. |
| **53 Faith/religion as a coping strategy** | "I'm a Christian and I pray in the morning, I worship, I’m thanks giving to God for what I got." (400 103) | -  *Contextual, not CGM acceptability* | **Social/Professional Role and Identity  Emotions  Behavioural Regulation** | Defines the participant’s Social/Professional Role and Identity (identity as a Christian) and serves as a primary mechanism for Emotions (worship and thanksgiving as affective regulation). It also relates to Behavioural Regulation as a self-initiated morning routine that provides structure and stability. |
|  | | | | |
| ***Theme 3: The CGM experience*** | | | | |
| **1 Issues with sensor adhesive** | "After she left, I went to the sink and I washed my arm. I washed my arm and I thought I would have to put some cream on, if there were any problems or infection, but there weren't. I didn't put any cream on. I just washed it and it was okay." (400 103)   "No, before it fell off I could tell that it wasn't sticky." (200 105) | **Affective Attitude  Burden  Perceived Effectiveness** | **Environmental Context and Resources  Beliefs about Consequences** | The physical properties of the intervention (Environmental Context and Resources) directly affect the Burden required to maintain the device or manage the skin post-wear. Participants evaluate the Perceived Effectiveness of the adhesive while formulating Beliefs about Consequences regarding potential side, shaping their Affective Attitude toward the intervention. |
| **2 Sensor came off prematurely** | "I had rung [name] on the Monday morning and said, it has come off, I am really sorry. [...] He was like, no, that's fine, there will still be obviously a week and a half's data on it." (500 104)   "The first time it came completely off I just lifted, I'd got a hoodie on [...] I just lifted it off over my head." (200 105)   "I slightly knocked the doorframe ... knocked my arm on the doorframe and it just fell off. [...] It wasn't even a hard knock, to be honest." (600 100) | **Perceived Effectiveness  Burden** | **Environmental Context and Resources  Emotions** | The technical failure of the sensor within the Environmental Context and Resources creates a physical Burden that complicates intervention participation. While the premature detachment is a significant event requiring an emotional response (Emotions) to the device failure , the intervention retains Perceived Effectiveness as long as the primary purpose of data collection is still met. |
| **8 DIY approach to getting the sensor to stick** | "...I was saying then, it is coming a bit loose and I don't know what to do. [...] I very stupidly said, I have got some Gorilla glue, he just went, no, no, no. When I next I spoke to her she went, you didn't use Gorilla glue did you?" (500 104)   "We got the monitor fitted, bandaged it round and I said, that’s going nowhere." (200 105) | **Burden  Self-efficacy** | **Environmental Context and Resources  Skills** | The Burden associated with ensuring the sensor remains attached leads to participants modifying their Environmental Context and Resources by using external materials like bandages. High Self-efficacy regarding intervention participation is manifested through the development of "DIY" coping strategies (Skills) intended to overcome perceived device failures. |
| **33 Experience of sensor wear** | "I thought I'd feel it more. Because as I said, most of the time I forgot it was there. It didn't hurt or anything or get in the way. It stuck on for the two weeks." (300 100)   "Honestly, I forgot it was even there." (400 103)   "No, only the fact that I'd forget I'd got it on, that's how come, the first time I wore it, that's how come when I pulled my top off quickly it's pulled it off." (200 105)   "No side effects, no pain, it's easy to wear you can take a shower or bath, whatever you want." (400 102) | **Affective Attitude  Burden** | **Environmental Context and Resources  Memory, Attention, and Decision Processes** | The minimal physical Burden and positive Affective Attitude towards sensor wear is facilitated by the device acting as a non-intrusive material resource (Environmental Context and Resources). This lack of physical discomfort reduces the requirement for selective focus (Memory, Attention and Decision Processes), often resulting in participants forgetting the intervention is in place until prompted by environmental interaction. |
| **35 Experience of sensor removal** | "And it came off easy." (300 103)   "I know it can be a bit painful having sticking plasters removed, but it wasn't anything like as bad as... I've had sticking plasters that have been more painful when they're removed than the glucose monitor. It was fine." (200 102) | **Affective Attitude  Burden** | **Environmental Context and Resources  Beliefs about Consequences** | The negligible Burden and positive Affective Attitude regarding sensor removal are linked to the properties of the adhesive (Environmental Context and Resources). Participants contrast this experience with negative expectations (Beliefs about Consequences). |
| **44 Experience of sensor fitting** | "The click of the machine actually is more scarier than the actual putting it into your arm. You can't feel it. It's just the click of a machine what scares you, putting it in. You just think, oh my goodness, that's loud, you know what I mean? That's loud." (400 103)   "No, it was alright. I'm okay with needles, so it was okay." (600 100) | **Affective Attitude  Burden** | **Emotions  Beliefs about Capabilities** | The positive Affective Attitude and minimal physical Burden of fitting ("can't feel it") are mediated by high self-confidence regarding needle-based procedures (Beliefs about Capabilities). Conversely, negative emotional reactions to the applicator noise ("scary") represent an Emotions domain influence that shapes the initial experience of the intervention. |
|  | | | | |
| ***Theme 4: Engagement with CGM as a strategy for diabetes management*** | | | | |
| **3 Interested in seeing CGM data** | "I’m looking forward to seeing what the results are." (300 100)   "Interviewer: Would you like me to send you a summary of the data that the sensor has collected from you? Respondent: Yes, please." (100 102) | **Affective Attitude** | **Knowledge  Beliefs about Consequences** | Affective Attitude (positive interest and anticipation) maps to Knowledge (desire for self-awareness) and Beliefs about Consequences (valuing the feedback provided by the device). This highlights high acceptability regarding the intervention's output. |
| **4 Sense of responsibility for the sensor** | "In fact the morning after I had it fitted I was in the shower, and of course my hand went on it and I thought, what the hell is that, oh it’s my glucose monitor. So, yeah." (300 100)   "I’d every intention of keeping it on two weeks" (200 105)   "Respondent: Yes. I miss it already. I keep checking for it. Interviewer: Aw, did you get used to it so much? Respondent: Yes." (100 102) | **Burden  Affective Attitude** | **Emotion  Intentions  Memory, Attention and Decision Processes** | Burden (the cognitive effort of being mindful of the device) and Affective Attitude (integration into daily routine/missing it) map to Emotion (adjustment) and Memory, Attention and Decision Processes (habitual checking). One quote highlights a high level of Intention toward adherence. |
| **22 Motivation for trying CGM** | "I wanted to help with research, but also, I've got a problem with blood sugars, so I wanted to help myself, but I also wanted to help them research that as well." (400 103)   "Interviewer: So when you were asked if you wanted to wear it, why did you agree? What were your reasons for saying yes? Respondent: Curious." (100 102)   "Might have been good for me, do you know what I mean, really good" (300 103) | **Ethicality  Perceived Effectiveness  Affective Attitude** | **Social/Professional Role and Identity  Beliefs about Consequences** | Ethicality (helping research) links to Social/Professional Role and Identity (role as a contributor). Perceived Effectiveness (expected personal benefit) maps to Beliefs about Consequences (outcome expectancies). Affective Attitude captures the "curiosity" regarding the device. |
| **23 Feelings about CGM prior to fitting** | "Interviewer: Were you nervous about it at all before she fitted it, were you? Respondent: No. I’ve got used to needles now, I have a lot of needles." (200 105)   "Interviewer: And before you had the sensor fitted, how did you feel? Respondent: Apprehensive at first. I was really thinking it’s going to hurt" (400 103) | **Affective Attitude** | **Emotion  Beliefs about Consequences** | Affective Attitude (feelings prior to the intervention) maps to Emotion (apprehension) and Beliefs about Consequences (anticipation of pain). Existing medical routines ("used to needles") mitigate prospective anxiety. |
| **24 Suggestions for sensor/CGM improvement** | "I think if I did it myself again I'd put it a bit further round my arm; it was towards the back of my arm instead of the front of my arm. So, Id reposition it a bit more." (300 100)   "Double sided tape as a cushioning effect. I don't know how it would be on your skin but you see the way it's set up now, it's very rigid towards flexible skin. [...] Now if you had double sided tape it's cushioned. [...] It's got stretch to it and it's cushioned so if it did bend, if the monitor did bend one way slightly it will absorb, the cushioning will absorb the effect of the movement." (200 105) | **Burden** | **Environmental Context and Resources  Skills** | Burden (physical/procedural effort) links to Environmental Context and Resources (material properties of the device) and Skills (placement technique). Suggestions focus on technical and placement modifications to optimize the device-body interface. |
| **25 Interested in unblinded sensor and app** | "Interviewer: You’ve already mentioned you’re using your phone and scanning it yourself, so is that something you’d be interested in trying out then? Respondent: Yes." (300 100) | -  *Relates to future use of unblinded CGM* | **Intentions  Knowledge** | Maps to Intentions (readiness to use the technology) and Knowledge (awareness of specific device functions). |
| **34 Feelings about wearing the sensor in retrospect** | "I’m fine now. I’m fine now. I’m glad I had it done." (400 103)   "It was a bit nervous having it on, but I got… it was great having it on. Yeah." (300 103)   "That I was contributing to research that could come in useful for others and the fact that wearing it doesn’t affect you in any way." (200 102) | **Affective Attitude  Ethicality  Burden** | **Emotion  Social/Professional Role and Identity  Beliefs about Consequences** | Affective Attitude (feeling glad/great) and Ethicality (contributing to research) map to Emotion and Social/Professional Role and Identity. Burden (low interference) maps to Beliefs about Consequences. Overall, retrospective acceptability is high, with altruistic motivations and low physical impact outweighing initial nervousness. |
| **36 Potential of unblinded CGM for diabetes management** | "It's a lot easier. A lot more reassuring having it on." (300 100)   "Interviewer: [...] Do you think this could manage your diabetes? R: Yes. [...] Interviewer: How do you think it could help? R: Not sure." (100 102) | -  *Relates to future use of unblinded CGM* | **Emotion  Beliefs about Consequences  Knowledge** | Maps to Emotion (reassurance), Beliefs about Consequences (perceived utility), and Knowledge (uncertainty regarding how the device functions). |
| **40 Prior knowledge of CGM** | "Yeah, I've seen a few people with them on. I wondered what they were. And I never knew until Nikita was dancing on Strictly Come Dancing; he took his shirt off and he had one, and then it was in social media about it. I thought oh, that?s what that is then." (300 100)   "No, never. [...] I had never heard of them." (500 104) | **Intervention Coherence** | **Knowledge  Social Influences** | Intervention Coherence (understanding the intervention) maps to Knowledge (awareness of existence) and Social Influences (modelling via media). Quotes illustrate knowledge gained through social modelling, as well as identifying a baseline knowledge gap. |
| **41 Benefits of CGM over SMBG** | "instead of having to do the intrusive jab yourself with a needle several times a day and then take the reading, you could do away with that because you’d just have that little discreet patch which is - it’s not more obtrusive or indeed intrusive than wearing a patch to try and stop you smoking. So it’s no more than like a nicotine patch or something like that." (200 102) | **Affective Attitude  Burden** | **Environmental Context and Resources  Social/Professional Role and Identity** | Replacing the "intrusive jab" reduces Burden, mapping to Environmental Context and Resources stressor. The "discreet" and non-obtrusive nature of the device fosters a positive Affective Attitude , mapping to Social/Professional Role and Identity by reducing potential social stigma or interference with one's self-presentation. |
| **42 Perception of CGM as a medical device** | "Interviewer: Did it feel at all like you were wearing a medical device? Respondent: No, not at all." (300 100)   "Interviewer: When you were wearing it, did it feel like you were wearing a medical thing? Respondent: Yes, it did." (100 102) | **Affective Attitude** | **Social/Professional Role and Identity** | Affective Attitude (emotional response to the device's nature) links to Social/Professional Role and Identity. The quotes illustrate varying degrees of conflict between the device's "medical" identity and the participant's self-perception. |
| **45 Effect of sensor on sense of self/own identity** | "Interviewer: So it didn't change the way [...] you felt about your body or yourself? Respondent: No." (100 102) | **Affective Attitude** | **Social/Professional Role and Identity** | Affective Attitude (neutral emotional response) maps to Social/Professional Role and Identity, indicating that wearing the CGM did not alter the participant's self-perception or body image. |
| **47 Understanding of glucose management** | "I thought, well, I wish I had known before that the exercise was what was making a bigger difference." (500 104)   "Interviewer: And what do you do when you've got a high reading? Respondent: I go a little bit walk and drink a lot of water." (400 102) | -  *Contextual, not CGM acceptability* | **Knowledge  Beliefs about Consequences** | Maps to Knowledge (understanding glucose drivers) and Beliefs about Consequences (experiential links between behavior and glucose levels). |
| **49 Reaction from others to CGM** | "I said to people I’m going to be wearing this fancy-pants glucose monitor. [...] People I told just wanted to see it." (300 100)   "People I told just wanted to see it. They wanted to know if it hurt or it got in the way or anything. I said I forget it’s there half the time." (300 100) | **Affective Attitude** | **Social Influences** | Affective Attitude (positive/neutral feeling about the device's visibility) links to Social Influences (interpersonal interactions and curiosity from others). The participant’s comfort in sharing the device reflects high social acceptability. |
